# Effect of the Communities That Care Prevention System on Adolescent Handgun Carrying: A Cluster-Randomized Clinical Trial

**DOI:** 10.1001/jamanetworkopen.2023.6699

**Published:** 2023-04-06

**Authors:** Ali Rowhani-Rahbar, Sabrina Oesterle, Emma L. Gause, Margaret R. Kuklinski, Alice M. Ellyson, Julia P. Schleimer, Kimberly Dalve, Elizabeth H. Weybright, John S. Briney, J. David Hawkins

**Affiliations:** 1 Department of Epidemiology, University of Washington, Seattle; 2 School of Social Work, Arizona State University, Tempe; 3 Social Development Research Group, School of Social Work, University of Washington, Seattle; 4 Department of Pediatrics, University of Washington, Seattle; 5 Department of Human Development, Washington State University, Pullman

## Abstract

**Importance:**

There is little information on upstream community-based interventions that reduce the prevalence of handgun carrying among adolescents, especially those growing up in rural areas.

**Objective:**

To test whether Communities That Care (CTC), a community-based prevention system focusing on risk and protective factors for behavioral problems early in life, reduces handgun carrying prevalence among adolescents growing up in rural areas.

**Design, Setting, and Participants:**

Community-randomized trial of 24 small towns in 7 states assigned randomly to the CTC or control group with outcomes assessed from 2003 to 2011. Participants were youths attending public schools in grade 5 who received consent from their parents to participate (77% of the eligible population) and were repeatedly surveyed through grade 12 with 92% retention. Analyses were conducted from June to November 2022.

**Interventions:**

A coalition of community stakeholders received training and technical assistance to install CTC, used local epidemiologic data to identify elevated risk factors and low protective factors for adolescent behavioral problems, and implemented tested preventive interventions for youth, their families, and schools.

**Main Outcomes and Measures:**

Handgun carrying (never vs at least once) operationalized in 2 ways: (1) prevalence of past-year handgun carrying, and (2) cumulative prevalence of handgun carrying from grade 6 through grade 12.

**Results:**

Overall, the 4407 study participants’ mean (SD) age was 12 (.4) years in both CTC (2405 participants) and control (2002 participants) communities in grade 6; about one-half of participants in each group were female (1220 [50.7 %] in the CTC group and 962 [48.1%] in the control group). From grade 6 through grade 12, 15.5% of participants in CTC communities and 20.7% of those in control communities reported carrying a handgun at least once. Youths in CTC communities were significantly less likely to report handgun carrying at a given grade than those in control communities (odds ratio [OR], 0.73; 95% CI, 0.65-0.82). The most pronounced effects were observed in grade 7 (OR, 0.70; 95% CI, 0.42-0.99), grade 8 (OR, 0.58; 95% CI, 0.41-0.74), and grade 9 (OR, 0.65; 95% CI, 0.39-0.91). Cumulatively from grade 6 through grade 12, youths in CTC communities were significantly less likely to report handgun carrying at least once than those in control communities (OR, 0.76; 95% CI, 0.70-0.84). Overall, CTC reduced the prevalence of past-year handgun carrying by 27% at a given grade and by 24% cumulatively through grade 12.

**Conclusions and Relevance:**

In this study, CTC reduced the prevalence of adolescent handgun carrying in participating communities.

**Trial Registration:**

ClinicalTrials.gov Identifier: NCT01088542

## Key Points

**Question:**

Does Communities That Care (CTC), a community-based prevention system focusing on shared risk and protective factors for behavioral health problems early in life, reduce the prevalence of handgun carrying among adolescents growing up in rural areas?

**Findings:**

In this community-randomized trial that enrolled 4407 youths in grade 5 and repeatedly surveyed them through grade 12, CTC reduced the prevalence of past-year handgun carrying by 27% at a given grade and by 24% cumulatively through grade 12.

**Meaning:**

In this study, CTC demonstrated prevention effects on handgun carrying among adolescents, suggesting that upstream interventions may reduce the burden of firearm injury among youths.

## Teaser

This cluster-randomized trial evaluates the effect of universal school-, family-, and community-based programs on adolescent handgun carrying.

## Introduction

Firearm injury is the leading cause of death among children and adolescents in the US.^1^ In 2020, 4076 adolescents aged 11 to 19 years died due to firearm injury. Of those deaths, the majority (64%) were homicides followed by suicides (32%).^2^ The burden of firearm death among communities across broad categories of the urban-rural continuum is not notably different. In 2020, for example, the rate of firearm death among adolescents in both metropolitan and nonmetropolitan areas was about 11 per 100 000.^2^

Handgun carrying is a key risk factor for youth violence in urban settings.^3^ Much less is known about adolescent handgun carrying in rural settings, which have sociocultural differences compared with urban settings.^4,5^ Handgun carrying may occur for sporting and recreational purposes in rural areas more frequently than it does in urban areas; however, evidence indicates that adolescent handgun carrying is associated with violence perpetration and exposure to violence in rural areas, too.^6,7^ Additionally, adolescents who carry handguns are more likely to report suicidal ideation and attempts than those who do not carry handguns, which is particularly salient in rural areas due to high rates of suicide.^8^

With some exceptions, federal law prohibits the purchase and possession of handguns by any person younger than 18 years.^9^ However, national data indicate that a sizeable number of adolescents carry a handgun. According to the National Survey on Drug Use and Health,^10^ the prevalence of past-year handgun carrying among adolescents aged 12 to 17 years was 4.6% in 2015 to 2019. Handgun carrying was especially prevalent among adolescents in nonmetropolitan areas (5.1%) compared with those in smaller metropolitan (3.9%) and larger metropolitan (3.1%) areas.^10^ Therefore, considering its scope and association with violence and injury, adolescent handgun carrying presents an opportunity to prevent firearm-related harm in rural and urban areas alike.

In recent years, calls for devoting resources to community-driven approaches for preventing firearm-related harm have notably increased.^11^ There exist evidence-based programs with demonstrated impact on various forms of youth violence^12^; however, fewer studies have specifically examined the effect of interventions on youth firearm carrying.^3,13,14,15^ These studies have been conducted in urban areas and among youths who are already involved in the cycle of violence. There is a striking paucity of information on universal, upstream, community-based interventions implemented earlier in life that could reduce the occurrence of high-risk firearm behavior, such as handgun carrying, among adolescents. Intervening at earlier developmental stages to reduce risk factors and strengthen protective factors for behavioral health problems may translate to lifelong improvements in health.

Communities That Care (CTC) is a science-based approach that activates a coalition of stakeholders to choose and implement tested and effective evidence-based interventions (EBIs) tailored to identified needs in the community with the overall objective of achieving collective positive impacts on youth development.^16^ The CTC prevention system reaches this goal by specifically increasing the use of EBIs that address elevated risk factors and low protective factors for adolescent problem behaviors prioritized by the community according to epidemiologic data from local youths.^17^ CTC builds prevention capacity in communities and provides a structure and process for community coalitions to adopt and implement EBIs based on local priorities. Evidence from a community-randomized trial has shown that communities that do not use CTC implement fewer EBIs and reach fewer children and families than those that adopt CTC.^18,19,20^

Prior studies have demonstrated the impact of CTC on reducing various health-risking behaviors such as drinking alcohol, smoking cigarettes, using drugs, and broadly engaging in any of several delinquent (eg, damaging property) or violent (eg, beating up someone) behaviors across different developmental stages from early adolescence through young adulthood.^21,22,23^ However, the effect of CTC specifically on handgun carrying has not been investigated. Examining this effect is important due to the role of handgun carrying in firearm violence among adolescents.^3^ Prior research has demonstrated some distinct trends, causal pathways, and consequences for firearm violence vs nonfirearm violence.^24,25^ As such, investigating whether community-wide preventive efforts focused on a broad range of shared risk and protective factors for behavioral health problems early in life affect handgun carrying, a key behavioral determinant of firearm violence specifically, is essential for informing the prevention of this type of violence. In this study, we tested the effect of CTC on handgun carrying among youths growing up in rural areas.

## Methods

This study follows the Consolidated Standards of Reporting Trials Extension (CONSORT Extension) reporting guideline. The University of Washington institutional review board approved the study protocol which is available in Supplement 1. Parental consent and student assent were obtained for all participants (77% of the eligible population) and did not differ between those in CTC communities and control communities.^26^

### Study Population and Intervention

**Figure 1.**
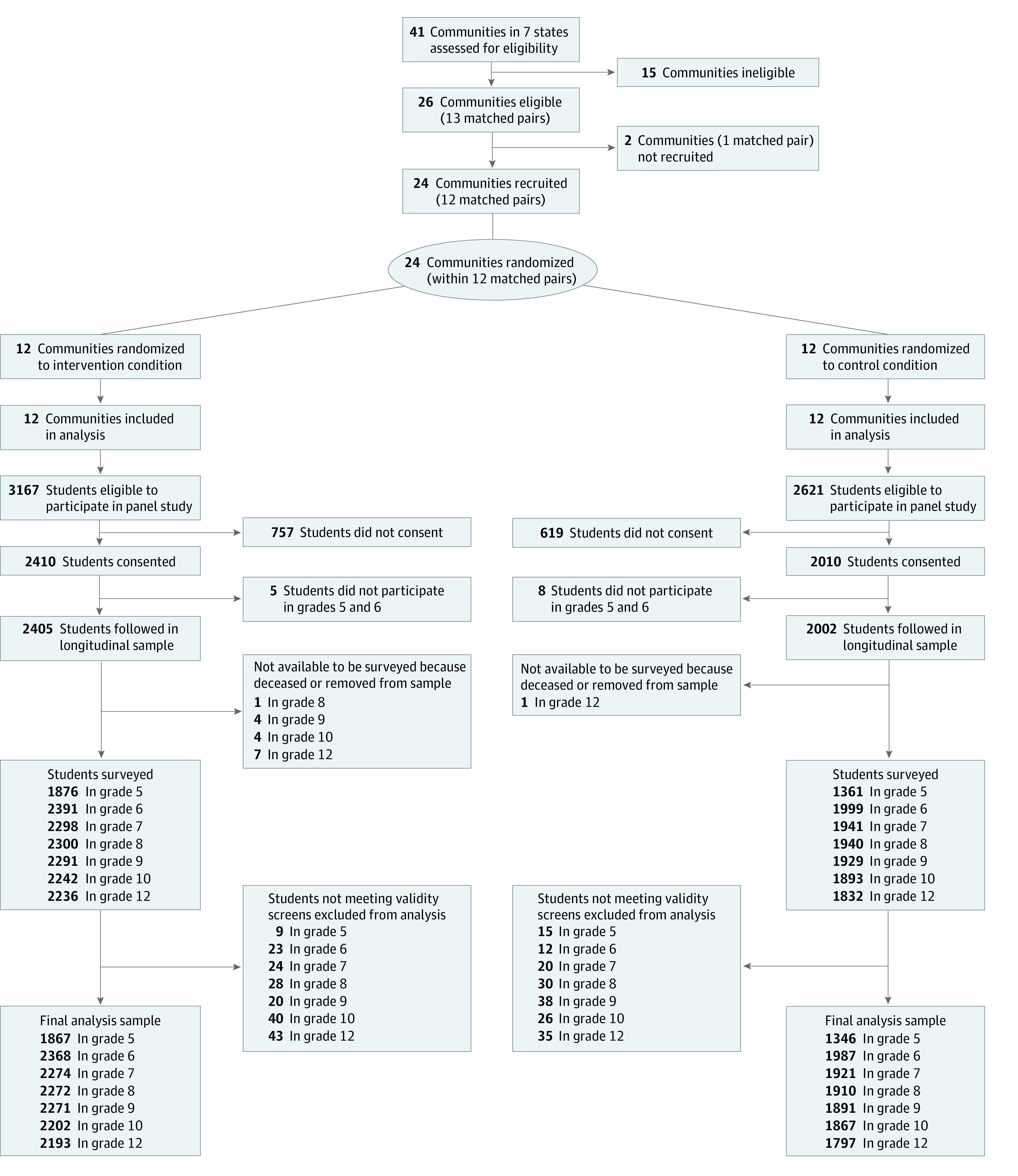
Flow Diagram of the Communities and Participants in the Randomized Trial

The sample included all 4407 youths who participated in the Community Youth Development Study (CYDS), a community randomized trial of the CTC prevention system beginning in 2003. This was a secondary analysis of the trial data through 2011. CYDS was powered to detect differences in youth risk and protective factors equivalent to an effect size of 0.25. It included 24 communities across 7 states (Colorado, Illinois, Kansas, Maine, Oregon, Utah, and Washington) whose mayors, city managers, school superintendents, and lead law enforcement officers agreed to participate in the study. Communities were rural incorporated towns with distinct geographic boundaries and governmental, educational, and law enforcement structures, with population sizes ranging from 1500 to 50 000 residents. The 24 communities were matched within state into 12 pairs (2-4 communities per state, yielding 1-2 pairs per state). Communities within each state that collectively had the closest values on population size, racial and ethnic diversity, economic factors, and crime rates were paired. Randomization of 1 member of each matched pair into the intervention condition and the other into the control condition occurred by coin toss (Figure 1). CYDS followed a longitudinal, grade cohort of public school students who were in grade 5 during the 2003 to 2004 school year in those communities. Recruitment continued into grade 6 to increase study participation.

CTC communities each identified locally specific elevated risk factors and low protective factors for adolescent problem behaviors in individual, peer, family, school, and community domains according to survey data from students in grade 6, 8, 10, and 12 in those communities. Although CTC was designed for children and youths ages 0 to 18 years, CYDS communities were asked to focus their prevention plans on programs for youths ages 10 to 14 years and their families and schools so that possible effects on drug use and delinquency could be observed within the initial 5-year study period. Starting with the 2004 to 2005 school year and annually thereafter, community coalitions implemented between 1 and 5 preventive programs to address their prioritized risk and protective factors. Overall, 18 different universal school-, family-, and community-based programs were implemented with high fidelity across the 12 intervention communities.^27^ The intervention communities selected different prevention programs to implement. These strategies included parent training programs (eg, group-based and self-administered programs), after-school programs (eg, skills-based interventions, mentoring, and tutoring services), and school-based programs (eg, drug prevention curricula and schoolwide organizational change strategies). The implementation fidelity for these programs was assessed via measuring adherence, dosage, quality of delivery, and participant engagement and was found to be high.^18,28^ CYDS repeatedly collected survey data from the longitudinal panel about risk, protection, behavior, and sociodemographic correlates at approximately ages 12 (grade 6), 13 (grade 7), 14 (grade 8), 15 (grade 9), 16 (grade 10), and 18 (grade 12) years with 92% or greater retention at each wave through grade 12 in 2011.^17,21,23,26^

### Outcome Measures

Handgun carrying was assessed from grade 6 through grade 12 by asking participants: “How many times in the past year (12 months) have you carried a handgun?” with ordinal response options of never, 1 to 2 times, 3 to 5 times, 6 to 9 times, 10 to 19 times, 20 to 29 times, 30 to 39 times, or 40 or more times. In all analyses, responses were dichotomized so that 0 indicated never carrying a handgun in the past year and 1 indicated carrying a handgun 1 or more times in the past year.

### Covariates

Individual-level covariates at baseline measured via self-report by youths included age, binary indicators for sex (female vs male), race (White vs minoritized racial and ethnic group [American Indian or Alaska Native, Asian, Black or African American, Native Hawaiian or Pacific Islander]), ethnicity (Hispanic vs non-Hispanic), maximum parental education (at least college completion vs less than college completion for either parent), attendance at religious services (almost weekly vs never/rarely), and a continuous scale measuring rebelliousness (mean of the following 3 items with a Cronbach α = 0.69: “I like to see how much I can get away with;” “I ignore rules that get in my way;” and “I do the opposite of what people tell me, just to get them mad” with responses as very false = 1 to very true = 4). Information on race was collected to examine the balance between the two groups with regard to its distribution. Examining race could inform equity-centered approaches to reduce the risk of firearm-related harm. Community-level covariates at baseline included the total population of students in the community and the percentage of students eligible for free or reduced-price school lunch.

### Statistical Analysis

 All analyses were conducted using an intent-to-treat framework on 10 multiply imputed data sets, and results were pooled across imputed data sets using Rubin rules.^30^ In primary analyses, we examined the effect of CTC on handgun carrying prevalence among CYDS participants from grade 6 through grade 12. This examination was conducted in 2 ways due to the episodic nature of handgun carrying demonstrated in the literature and in our prior studies in this population.^3,4,5^ Missing data were imputed using multivariate imputation by chained equations in R statistical software version 4.0.0 (R Project for Statistical Computing).^29^ Statistical significance level was set at .05. Data were analyzed from June through November 2022.

In primary analysis 1, the prevalence of past-year handgun carrying was longitudinally examined over time. In this analysis, we used mixed effects logistic regression models with responses over time clustered within individuals and standard errors clustered within randomized communities using sandwich estimators. A series of binary indicators was included to account for the nesting of the 24 communities in 12 pairs, and a series of binary indicators for grades was included to index the wave of repeated measures. We also estimated grade-specific effects of CTC by interacting the CTC intervention indicator with the grade indicators in these models.

In primary analysis 2, cumulative prevalence of handgun carrying from grade 6 through grade 12 was examined. In this analysis, the outcome was whether participants ever (vs never) reported handgun carrying between grades 6 and 12, and the regression models did not include the grade indicators for repeated measures.

In CYDS, no information on handgun carrying was collected in grade 5; the earliest information on handgun carrying was collected in grade 6. As such, we conducted several sensitivity analyses to examine the robustness of findings in primary analysis 1 (ie, prevalence of past-year handgun carrying). In sensitivity analysis 1, we shifted the analysis forward by 1 year so grade 6 could be used as the baseline. The rationale for this analysis was twofold. First, as previously mentioned, recruitment continued into grade 6 to increase study participation. Second, even youths recruited in grade 5 had received very little prevention programming by grade 6. In sensitivity analysis 2, we repeated sensitivity analysis 1 only among youths who did not report handgun carrying in grade 6. In sensitivity analysis 3, we adjusted for delinquency in grade 5 as a proxy measure for handgun carrying. Of note, there was baseline equivalence between CTC communities and control communities in delinquency in grade 5.^31,32^ In sensitivity analysis 4, we repeated sensitivity analysis 3 only among youths who did not report delinquency in grade 5. Additionally, we conducted a sensitivity analysis (sensitivity analysis 5) for primary analysis 2 (ie, cumulative prevalence of handgun carrying) in which cumulative prevalence of handgun carrying through grade 12 was examined only among youths who did not report handgun carrying in grade 6.

## Results

**Figure 2.**
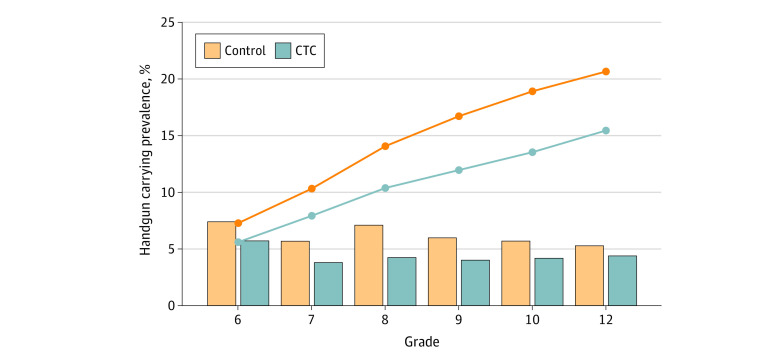
Percentage of Participants Who Reported Handgun Carrying in Each Grade (Bars) and Cumulatively From Grade 6 Through Grade 12 (Lines) by Communities That Care (CTC) Intervention Status

The mean (SD) age of study participants in grade 6 was 12.08 (0.40) years in the CTC communities and 12.09 (0.39) years in the control communities. About half of participants in both CTC communities and control communities were female (1220 [50.7%] in the CTC group and 962 [48.1%] in the control group) (Table). The prevalence of handgun carrying in the past year at each grade (ie, study wave) ranged from 3.8% (86 participants) to 5.7% (135 participants) in CTC communities and from 5.3% (95 participants) to 7.4% (146 participants) in control communities. From grade 6 through grade 12, 15.5% (372 participants) of participants in CTC communities and 20.7% (414 participants) of those in control communities reported carrying a handgun at least once (Figure 2).

**Table.** Characteristics of Participating Youths and Communities by Intervention Status^a^

Characteristic	No. (%)	
	CTC (n = 2405)	Control (n = 2002)
Individual-level characteristics		
Age, mean (SD)^b^	12.08 (0.40)	12.09 (0.39)
Sex		
Female	1220 (50.7)	962 (48.1)
Male	1185 (49.3)	1040 (51.9)
Race^c^		
American Indian or Alaska Native	152 (6.3)	116 (5.8)
Asian	32 (1.3)	43 (2.1)
Black or African American	102 (4.2)	67 (3.3)
Native Hawaiian or Pacific Islander	20 (0.8)	15 (0.7)
White	1835 (76.3)	1310 (65.4)
Missing	12 (0.5)	19 (0.9)
Other^d^	391 (16.3)	546 (27.3)
Ethnicity		
Hispanic	363 (15.1)	532 (26.6)
Non-Hispanic	2042 (84.9)	1470 (73.4)
Parents’ maximum education^e^		
Grade school or less	47 (2.0)	78 (3.9)
Some high school	135 (5.6)	162 (8.1)
Completed high school	408 (17.0)	409 (20.4)
Some college	547 (22.7)	466 (23.3)
Completed college	829 (34.5)	555 (27.7)
Graduate or professional degree	339 (14.1)	239 (11.9)
Missing	100 (4.2)	93 (4.6)
Attendance at religious services		
Never	426 (17.7)	389 (19.4)
Rarely	564 (23.5)	533 (26.6)
1-2 Times a month	328 (13.6)	278 (13.9)
Once a week or more	1004 (41.7)	738 (36.9)
Missing	83 (3.5)	64 (3.2)
Rebelliousness scale score, mean (SD)^f^	1.6 (0.65)	1.6 (0.68)
Community-level characteristics		
No. of enrolled students in the community, mean (SD)	3582.05 (1856.98)	3839.39 (2473.87)
Percentage of students eligible for free/reduced lunch in the community, mean (SD)	38.03 (11.36)	41.34 (12.92)

Abbreviation: CTC, Communities That Care.

^a^ Information presented in the table pertains to grade 6.

^b^ Information was missing for 38 participants in CTC communities and 14 participants in control communities.

^c^ Categories are not mutually exclusive.

^d^ Overall, 62.4% and 78.8% of participants who chose other race in CTC and control communities reported Hispanic ethnicity, respectively. Other was a write-in category with no predetermined list of races.

^e^ For either parent.

^f^ Information was missing for 51 participants in CTC communities and 32 participants in control communities. The range for this scale was from 1 to 4.

**Figure 3.**
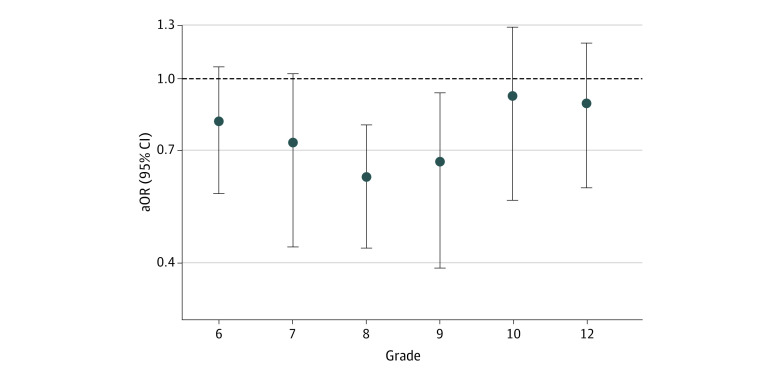
Effect of Communities That Care Prevention System on Prevalence of Handgun Carrying by Grade Odds ratios are adjusted for individual (age, sex, race, ethnicity, maximum parental education, attendance at religious services, rebelliousness) and community (total population of students in the community and the percentage of students eligible for free or reduced-price school lunch) characteristics. aOR indicates adjusted odds ratio.

In adjusted analyses, participants in CTC communities were significantly less likely to report handgun carrying in the past year at a given grade than those in control communities (odds ratio [OR], 0.73; 95% CI, 0.65-0.82). The most pronounced effects were observed in grade 7 (OR, 0.70; 95% CI, 0.42-0.99), grade 8 (OR, 0.58; 95% CI, 0.41-0.74), and grade 9 (OR, 0.65; 95% CI, 0.39-0.91; Figure 3). Cumulatively from grade 6 through grade 12, participants in CTC communities were significantly less likely to report handgun carrying at least once than those in control communities (OR, 0.76; 95% CI, 0.70-0.84). The full results of primary analyses including coefficients for all covariates are included in eTable 1, eTable 2, and eTable 3 in Supplement 2. In all sensitivity analyses, the findings remained materially the same as those found in primary analyses with ORs for the CTC intervention effect ranging from 0.76 (95% CI, 0.66-0.86) to 0.80 (95% CI, 0.67-0.96) (eTable 4, eTable 5, eTable 6, eTable 7, and eTable 8 in Supplement 2).

## Discussion

Findings from this longitudinal community randomized trial indicate that the CTC prevention system significantly reduced the prevalence of handgun carrying among youths growing up in rural areas. Youths in CTC communities were less likely to report handgun carrying in each wave of the study and cumulatively from grade 6 through grade 12 than those in control communities.

The intervention effects were more pronounced in grades 7, 8, and 9, grades in which CTC communities were actively implementing CTC. As participants became older (ie, grades 10-12), the differences in the past-year prevalence of handgun carrying between youths in the 2 groups of communities diminished. It is important to note that in this trial, little preventive programming targeted the high school years, and few students in the longitudinal panel were exposed to tested and effective EBIs beyond grade 9.^17,21^ Still, youths in CTC communities were significantly less likely than those in control communities to report handgun carrying through grade 12 cumulatively.

In recent years, historic investments have been made to support community violence intervention (CVI) programs that focus on reducing shootings by establishing relationships with people at the center of firearm-related harm in communities.^33,34^ These programs support people at the highest risk of being survivors or perpetrators of violence (including those who have already sustained firearm injury) through street outreach, violence interruption, and case management.^35^ The Ceasefire Program in Oakland^36^; Cure Violence Program in New York,^37^ Chicago,^38^ and Philadelphia;^39^ and Gang Reduction and Youth Development Program in Los Angeles^40^ are examples of strategies that have been associated with varying degrees of reduction in risk of firearm violence. Alongside these valuable investments in CVI programs, there is also a need to move further upstream and evaluate strategies that could affect risk and protective factors earlier in life, with downstream prevention effects on high-risk firearm behavior through adolescence in various geographic settings including in rural communities. Such strategies could also garner broad policy support across the political spectrum.

The CTC prevention system presents a prime example of such strategies. CTC is a data- and community-driven prevention system designed to assist communities in selecting and implementing EBIs aligned with their strategic prevention priorities for improving the health of youths community-wide, which are based on local data identifying elevated risks and low protective factors in the community.^17^ EBIs can improve public health if implemented community-wide and with high fidelity; however, widespread implementation typically does not occur.^23^ This is partly because communities often lack implementation support systems. The CTC system was developed to provide this support based on theories of public health promotion, community competence, and prevention science.^41,42^

Findings of this study provide the first empirical evidence on the impact of CTC on handgun carrying among youths growing up in rural areas. This is especially important considering evidence on the association between adolescent handgun carrying and a multitude of behavioral indicators of exposure to violence and perpetration (eg, bullying, use of weapons to get money) in rural communities, some of which may involve the use of firearms specifically.^6^ Future investigations should examine the specific pathways by which CTC influences handgun carrying, assess the impact of CTC on firearm violence, and explore factors that may moderate these effects across different settings and subpopulations. Notably, considering the frequency of firearm use for sporting and recreational purposes in rural areas, it will be important to examine the impact of CTC on firearm violence among rural adolescents who carry firearms for reasons and in contexts that elevate the risk of violence.

### Limitations

This study had limitations. First, all analyses were based on self-report data which are subject to social desirability and recall biases. Of note, communities, not students, were randomized into intervention groups in this trial. It is unlikely that students in the study were aware of the intervention group to which their community belonged; thus, it is unlikely that there was differential self-report by intervention group that might account for observed effects. Second, the survey did not ask from whom (eg, family or friend) youths obtained the handgun, how they obtained it (eg, purchasing or renting), or why they carried it (eg, self-defense or retaliation). Third, communities studied were towns of 50 000 or fewer residents and were not necessarily representative of the entire country, specific regions, certain states, or rural communities not represented in the trial.

## Conclusions

Reducing firearm carrying among adolescents is an important national public health priority and one of the objectives of Healthy People 2030.^43^ We provide evidence that the CTC prevention system reduced the prevalence of handgun carrying among adolescents growing up in rural areas, up to 7 years after the system was first installed. These findings highlight the potential for effective community-based nonpunitive programs that influence risk and protective factors early in life to exert downstream prevention effects on high-risk firearm behavior and in turn firearm-related harm.

## Notes

Supplement 1.
Trial Protocol

Supplement 2. **eTable 1. **Findings of Primary Analysis 1 for Past-Year Handgun Carrying **eTable 2. **Findings of Primary Analysis 1 for Grade-Specific Past-Year Handgun Carrying **eTable 3. **Findings of Primary Analysis 2 for Cumulative Handgun Carrying **eTable 4. **Findings of Sensitivity Analysis 1 for Past-Year Handgun Carrying **eTable 5. **Findings of Sensitivity Analysis 2 for Past-Year Handgun Carrying **eTable 6. **Findings of Sensitivity Analysis 3 for Past-Year Handgun Carrying **eTable 7. **Findings of Sensitivity Analysis 4 for Past-Year Handgun Carrying **eTable 8. **Findings of Sensitivity Analysis 5 for Cumulative Handgun Carrying

Supplement 3.
Data Sharing Statement
