# Error in Figure 3

**DOI:** 10.1001/jamanetworkopen.2023.15284

**Published:** 2023-05-08

In the Original Investigation titled “Effect of the Communities That Care Prevention System on Adolescent Handgun Carrying: A Cluster-Randomized Clinical Trial,”^1^ published April 6, 2023, incorrect odds ratios were shown in Figure 3. These values were based on a sensitivity analysis rather than the main analysis. This article has been corrected.^1^
