# Supplementary material for: Effect of the Communities That Care Prevention System on Adolescent Handgun Carrying: A Cluster-Randomized Clinical Trial
Source: JAMA Netw Open. 2023 Apr 6;6(4):e236699. doi: 10.1001/jamanetworkopen.2023.6699 (PMC10080373; doi:10.1001/jamanetworkopen.2023.6699)
Supplement: Supplement 1. — Trial Protocol [file jamanetwopen-e236699-s001.pdf]

J. David Hawkins

SCIENCE-BASED PREVENTION:
TESTING COMMUNITIES THAT CARE PROTOCOL AND STATISTICAL ANALYSIS PLAN

Experimental Design and Methods

In 13 of the 20 matched pairs of communities participating in the Diffusion project, neither community has progressed in the use of prevention science to the point of using tested effective policies and programs to address risk and protective factors elevated in epidemiologic community data. In the Diffusion project, these communities were matched within each state on population size, economic indicators, and crime data. Twelve pairs of these communities (24 communities) have been recruited to participate in the proposed study. One member of each pair has been randomly assigned to the CTC intervention condition; the other member of each pair has been assigned to the control group.

The intervention component will begin in the fall of Year 1, with a community activation and education process using Communities That Care training events, materials, and technical assistance. (See Appendix C for examples of the CTC community training guides from Developmental Research and Programs, the training and technical assistance subcontractor.) Intervention communities will be trained to use the baseline data collected in Year 5 of the Diffusion project, just prior to the start of the proposed study, to prioritize specific areas and risk and protective factors for attention. By the ninth month of the project, communities will select effective interventions to address their prioritized needs and create strategic community prevention plans to implement these interventions. Selected policies and programs will be offered to affect participants in the panel sample in each community beginning in the Fall of Year 2 and continuing through Year 5 of the study.

Effects of the CTC intervention on risk factors, protective factors, substance use, and other problem behaviors will be assessed using repeated cross-sectional and longitudinal student surveys in both intervention and control communities. Implementation and impact of the CTC intervention on communities' prevention service systems will be assessed using the CKI telephone interviews of community key informants, and the CRD surveys of prevention service providers in all communities.

The Intervention

CTC is a system for changing the way communities plan and conduct prevention services. It provides training and tools for community activation, risk and resource assessment, and strategic prevention planning needed to implement and institutionalize risk- and protective-focused prevention. CTC changes the structure and process of prevention service planning and delivery. It empowers communities to approach prevention analytically, and it puts in place tools that communities can use continuously to monitor and inform their progress in reducing risks, enhancing protection, and preventing health and behavior problems. CTC is designed to lead to changes in communities' systems of prevention services that will reduce rates of adolescent health and behavior problems in communities that use the program.

The 12 communities assigned to the intervention condition will receive training and technical assistance in using CTC from Developmental Research and Programs (DRP) and from project staff. These communities also will receive annual subcontracts to hire a full-time project coordinator for the five years of the study and to support implementation of tested preventive interventions selected by the communities in the second through fifth years of the project. The proposed experimental test will focus interventions on children in elementary and middle schools (grades 5 through 8)

CTC consists of five phases designed to mobilize community leaders, and a community prevention board to plan and implement a set of tested interventions to reduce elevated risk factors and promote protective factors in the community. The CTC intervention is manualized, and includes training events and guides for community leaders and board members (see Appendix C), as well as technical assistance (Developmental Research and Programs, 1997).

<u>Phase I</u>. Phase I begins with a Community Readiness Assessment focused on attitudinal and organizational characteristics of community members, leaders, and organizations that influence the mobilization process (e.g., Arthur et al., 1996; National Institute on Drug Abuse, 1997; Oetting et al., 1995). In this study, readiness assessment will be informed by the results of the Community Key Informant interviews and Community Resource Documentation conducted during the second and fourth years of the Diffusion study. These data provide information on the degree to which community leaders, educators, and service providers understand, endorse, and use risk- and protective-focused prevention approaches, allowing CTC training and technical assistance activities to be tailored to each community's specific stage of readiness.

Phase II,. Phase II of the CTC intervention begins with one-half day of training to orient key community leaders (mayor, police chief, school superintendent, and business, faith, community, and media leaders) to risk and protection focused prevention and the community mobilization processes. Key community leaders are activated by CTC to shape opinion and direct resources to ensure that the programs and policies developed through the CTC process are fully implemented and institutionalized (Finnegan & Ervin, 1989). They also are expected to hold the community prevention board and staff accountable for planning and carrying out CTC. The training defines roles and responsibilities of the key leaders and those of the community prevention board. The key leaders make a commitment to actively support the project, hold the community board accountable, and maintain the board's visibility within the community.

Following the key leader training, the leaders in each community identify and invite about 30 community members to constitute a community prevention board. The community prevention board is the mechanism for carrying out the planning and implementation activities of CTC. Whether constituting a new board or using an existing coalition or board, key leaders seek to ensure that the board includes members who represent the diversity of the community who can assist in the development of linkages to resources, organizations, and agencies that can support the board's prevention work. The boards include leaders and opinion shapers in their respective sectors, leaders of grassroots constituencies, and school and agency representatives involved in delivering programs and services to children, families, and neighborhoods. In Phase II, community prevention board members attend a two-day orientation training, which provides an overview of the CTC intervention and information on risk and protective factors, the social development strategy, development or refinement of the group's vision for the future of the community's children, and strategies for developing and maintaining effective board organization.

Phase III, Develop a Data-Based Profile of Community Strengths and Challenges. Community prevention board members participate in a two-day training on how to utilize epidemiological data on risk and protective factors. Board members learn to interpret survey and archival social indicator measures of risk and protective factors. To accomplish the aims of this study during the five-year study period, community boards will focus on identifying risk and protective factors affecting youths in grades 5 to 8. The board will use these data to identify neighborhoods where youths are exposed to high levels of overall risk and low levels of protection during early adolescence, and to identify those risk factors that are most elevated and those protective factors most depressed in these areas, as well as in the community at large. (Examples of risk and protective factor profiles from three communities derived from the year 2000 student surveys in the Diffusion project are provided in Appendix F).

By comparing levels and trends in risk and protection with national data available from Developmental Research and Programs (DRP) and with state data available from the Diffusion project, boards will select specific areas within the community for concerted attention and will prioritize two to five specific risk factors for preventive action. These profiles of risk and protection will also provide baseline data for subsequent assessments of the community's progress in changing levels and trends in risk factors targeted by the board's prevention plans.

Following the profiling and prioritization of risk and protective factors affecting community youths during early adolescence, prevention board members attend a one-day resource assessment training. The training provides methods for assessing the community's existing prevention resources. The goal of this assessment is to identify gaps in existing policies, programs, and services that address the community's prioritized neighborhoods and risk factors. Gaps can include the unavailability of preventive programs and services in prioritized neighborhoods, the use of ineffective prevention programs or services, poorly implemented services, or poorly enforced policies. In this study, community resource assessment will be facilitated by the availability of data on community preventive policies and services from the Community Resource Documentation conducted in the second and fourth years of the Diffusion project.

Phase IV, Create a Comprehensive Youth Development Plan. In Phase IV, the CTC prevention board uses the prioritization done in Phase III to develop and implement a community prevention plan. The board defines clear, measurable, desired outcomes with respect to risk reduction, protection enhancement and health and behavior problem reduction goals. Specifying outcomes helps to clarify the policies, programs and activities that will be needed to achieve them. Community board members attend a two-day "Effective Prevention Strategies" training workshop that reviews tested policies, programs and actions that have been effective in reducing the community's prioritized risk factors and enhancing protective factors in adequately controlled trials. The board develops an action plan to fill

identified resource gaps through the implementation of tested, effective policies, programs, and actions.

To test the effects of CTC in achieving observable reductions in prioritized risk factors and drug use initiation within the five-year study period, the intervention communities will focus their strategic plans on interventions for elementary and middle school-aged youth. The communities will implement interventions targeting elementary school-aged youths (fifth and sixth grades) during the second year of the project, then will add interventions focusing on middle school-aged youths (sixth or seventh through eighth or ninth grades) during the third through fifth years of the project. The community prevention boards will select policies and programs from a menu of tested preventive interventions targeting elementary and middle school youths (see Table 1 and Appendix E), a subset of those included in "Communities that Care Prevention Strategies: A Research Guide to What Works," (DRP, 2000, see Appendix C). The interventions included in the menu for this test were selected using the following criteria: (a) Each has shown positive effects in reducing one or more risk factors, enhancing protective factors, and reducing drug use and/or related behavior outcomes in controlled experimental or quasi-experimental studies; (b) Training, technical assistance and manuals are available to guide communities in the installation of the policy or program; and (c) The policy or program affects risk, protection and behavior for youth aged 10 through 14.

Because change efforts are more likely to produce observable effects when prioritized risk and protective factors are addressed consistently across multiple socialization domains (Catalano et al., in press; Tobler et al., 2000), community boards will be expected to select at least three preventive interventions for implementation: a family based intervention, a school based intervention, and a community focused intervention. By selecting interventions in each domain, it is hypothesized that the new interventions will have a synergistic impact on the prioritized risk factors. For example, an important goal of the strategy is to strengthen norms against problem behavior in all three domains, thus creating clear and consistent behavioral expectations for youths in the community. The result will be a coordinated set of policies and programs designed to reduce the prioritized risks while enhancing protection.

The plans will describe the evidence-based prevention strategies selected to address prioritized risk and protective factors and will include work plans to implement these new strategies in the community. The plans will describe how each selected intervention will be implemented to reach the community's youths living in neighborhoods where overall risk exposure is high with mutually reinforcing risk reduction and protective factor enhancement activities affecting family, school and community. The plans will also specify how the interventions will be coordinated with existing programs and resources. The community prevention boards will complete their action plans by specifying plans for monitoring implementation quality and providing feedback to support continuous quality improvement (Wandersman et al., 1998), and for assessment of progress towards specified process and outcome goals. Project staff will assist in ensuring that training and technical assistance is accessible to intervention communities as they develop and implement their plans.

Table 1: Menu of Effective Interventions for Grades 4-8

Name of Effective Program (Grades 4-5)	Name of Effective Program (Grades 6-8)
Parent Training	
Preparing for the Drug-Free Years	Preparing for the Drug-Free Years
The Incredible Years: Parent's, Teacher's and Children's Videotape Series	Adolescent Transitions Program
Strengthening Families Program	Strengthening Families Program
The Iowa Strengthening Families Program	The Iowa Strengthening Families Program
Strengthening Families Program: for Parents and Youth 10-14	Strengthening Families Program: for Parents and Youth 10-14
Multidimensional Therapy<br>Brief Strategic Family Therapy<br>Functional Family Therapy	Multidimensional Family Therapy<br>Brief Strategic Family Therapy<br>Functional Family Therapy
Creating Lasting Connections	Creating Lasting Connections
Organizational Change in Schools	
School Development Program	The Program Development Evaluation (PDE) Method
Classroom Organization, Management, and Instructional Strategies	
The Good Behavior Game	Behaviorally-Based Prevention Program
Continuous Progress Instruction	Continuous Progress Instruction
Computer-Assisted Instruction	Computer-Assisted Instruction
Cooperative Learning Programs	Cooperative Learning Programs
Tutoring Programs	
Reading Recovery	
Classroom Curricula for Social & Emotional Competence Promotion	
Growing Healthy	Growing Healthy
Promoting Alternative Thinking Strategies (PATHS)	Adolescent Alcohol Prevention Trial (AAPT)
PeaceBuilders	Life Skills Training (LST) Program
Know Your Body	Know Your Body
The Children of Divorce Intervention Program	Reconnecting Youth Program
	Project Alert
	Alcohol Misuse Prevention
	All Stars
Multi-component Programs Based in Schools	
Midwestern Prevention Project/Project Star	Midwestern Prevention Project/Project Star
Success for All	Project Northland
Child Development Project	
Mentoring	
Big Brothers/Big Sisters	Big Brothers/Big Sisters
	Across Ages
Community Mobilization	
The Midwestern Prevention Project/Project Star	The Midwestern Prevention Project/Project Star
Community/School Policies	
Regulation of Availability	Regulation of Availability
Taxation	Taxation
Mandatory Sentencing Laws for Use of a Firearm during the Commission of a Felony	
Community Policing Strategies	Community Policing Strategies

Prior to funding the community boards' implementation plans, the plans will be reviewed by the project's Review Panel (comprised of project staff, the project's Advisory Board, and state substance abuse prevention officials from each of the collaborating states) and revised by the community boards

in light of the Review Panel's input. The collaborating state officials will contribute their knowledge of the local providers and community cultures. Project staff and Advisory Board members will contribute their knowledge of effective implementation of science-based preventive interventions. The resulting dialogue between the community prevention boards, state prevention officials, and researchers will ensure the completion of high quality, feasible plans that address each community's profile of risks and protection (e.g., Holder & Reynolds, 1998). Funding for proposed programs up to $75,000 annually will be determined by the Review Panel, and will be awarded and monitored by SDRG.

Phase V. Implement and Evaluate. After finalizing their action plans, the Boards will implement and evaluate their plans. A task force of the board will be created for each new intervention to be installed. Task force members will include program implementers and members of their support structure. For example, the task force to oversee implementation of a new school curriculum for social competence promotion will include at least one board member, teachers, a principal, a parent whose child attends the school where the program will be implemented, and other school support staff. Task force members will develop steps to achieve high quality program implementation and to implement process and outcome measures included in the action plans so that implementation can be monitored. DRP and project staff will provide training and technical assistance to ensure high quality implementation and monitoring of progress toward implementation and outcome goals. Beginning in Year 2 and continuing into Year 4, the community prevention boards will receive training to implement the evidence-based prevention strategies in their community prevention plans. Communities will contract for the specific training events required to implement their selected interventions with the organizations that provide this training. Follow-up training and ongoing technical assistance will be provided by the intervention coordinator and DRP to develop the capacity within the community to implement the strategy effectively, including removing barriers to implementation, marshalling resources, and developing procedures to monitor the implementation of each element (Chavis et al., 1992; Florin et al., 1993).

The proposed study is designed to achieve widespread community activation and intensive implementation of the selected interventions. Staffing and budgetary levels have been established to ensure that intervention communities will receive adequate resources to implement widespread, coordinated interventions in multiple social domains for four years. The school and family interventions implemented in the second project year will focus on elementary schools, while those implemented in Years 3 through 5 will target middle schools as the longitudinal panel moves from elementary to middle school grades. Resources are provided to serve 2 to 4 elementary/middle school populations identified through the school survey data as having the highest aggregate levels of risk and lowest levels of protection in the community. The resources will reach at least ten classrooms, 200 students, and 100 parents each year in each intervention community. In communities of less than 15,000 residents, the resources requested will be adequate to implement new interventions for all youths in the target ages, while in the larger communities youths attending those schools identified through risk assessment as serving populations at highest risk will receive the interventions.

In addition to implementing programs targeting elementary and middle school students and their families, each community board will mobilize broad sectors of the community to work to change community norms, policies and practices regarding adolescent problem behaviors through the task force structure described earlier. This task force will address community norms by affecting policy changes and by creating the informal social impetus to strengthen the impact of such changes. The board will also involve local media in mobilizing the community and generating widespread support for the board's efforts by publicizing their mission, objectives, decisions, and activities. Media will be used to: (a) educate community members about risk and protective factors for adolescent problem behaviors; (b) communicate clear norms and enforcement policies regarding problem behavior in the community; (c) generate public support for regulatory measures; and (d) motivate community members to take part in efforts to reduce risk factors and promote protective processes in the community. Representatives of the local media will be recruited to the board to provide expertise in these efforts.

The family and school interventions will thus be implemented within the context of a community where the formal and informal leaders have joined to establish a shared vision for the future of the community's children, where this vision is repeatedly presented to the entire community, and where there is an ongoing invitation to community members to support and participate in the efforts to move toward this vision. The combined interventions will focus on reducing specific risk factors prioritized by the community board through their risk and resource assessment and will target a limited age grouping of children (elementary and middle school students) and their families. These interventions are

expected to produce measurable community-wide effects on targeted risk factors among children aged 10-14, a developmental period of particular salience for problem behavior initiation.

Maintaining Responsiveness to the Community. An important task is to establish and maintain the community prevention boards as representative, visible, enduring, and responsive coalitions of community residents, formal and informal community leaders, and agency representatives. This will be accomplished by hiring and training a local full-time community coordinator to staff the board's day-to-day activities, and by the formation of board task forces. The community coordinators will be responsible for ensuring that the boards are inclusive of all groups within their communities and responsive to the diverse needs of these groups. Community coordinators will establish contacts with leaders of the various grassroots constituencies and racial and ethnic groups within their communities to solicit their involvement on the community board and to discuss their issues and concerns. The community coordinators will also review board activities and products with these informal leaders to ensure the appropriateness and acceptability of the boards' actions to all segments of the community. The community coordinators will be trained to involve a wide range of community members in the CTC initiative, and to provide the leadership, coordination, and incentive management needed to motivate board members to remain active (Parcel et al., 1989; Prestby et al., 1990). They will be trained each year to develop skills in program implementation and institutionalization, and to address mobilization and board maintenance issues.

Sample

Community selection. Thirteen pairs of communities participating in the Diffusion study were eligible for inclusion in this study, and 12 pairs have been recruited for the proposed study. These communities have demonstrated their commitment to participate in SDRG research by providing a variety of data for the Diffusion project. In all these pairs, neither community has progressed to the point of installing tested effective policies and programs to address prioritized community risk and protective factors. We have randomly assigned these matched communities to the intervention or control condition. The CTC intervention is expected to produce measurable changes in the prevention service systems in the communities which, in turn, are expected to affect levels of risk, protection, and outcomes among youth aged 9 to 14 over the study period.

The 24 communities in the study were matched with regard to size, poverty and crime indices for the Diffusion project. For the proposed study, this matching maximizes the baseline comparability between the experimental and control communities and reduces potential threats to study’s internal validity. Analysis of student survey data from the 1998 and 2000 survey administrations in these communities revealed no significant differences between the intervention and control communities with regard to levels of risk and protective factors or drug use outcomes. Table 2 provides descriptive data on the characteristics of the intervention and control communities

Including moderate-sized communities. The communities in the proposed study have a population between 1,500 and 50,000 residents with a clear community identity and boundaries. These are small- to moderate-sized towns with their own governmental, educational and law enforcement structures. Focusing on communities within this restricted size range will limit the generalizability of study findings, but there are important methodological advantages to using towns of this size in community studies (Biglan, 1995). These communities are small enough that their prevention service systems can be clearly defined and measured. Because the enrolled student population in a single grade ranges from 40 to 506 in these communities, with only two communities exceeding 400 students per grade, the study can expose a large proportion of the early adolescent youths in the intervention communities to the chosen programs and policies at a reasonable cost without compromising the power of the experiment. The use of small-to moderate-sized communities increases the likelihood of observing effects of changes in community prevention services on the levels of risk and protective factors experienced by youths in these communities.

Randomization. Randomization of one member of each pair of eligible communities occurred prior to recruitment into the proposed study. For each pair, a coin toss determined which of the two matched communities was assigned to the intervention condition.

Avoiding cross-community contamination. It is essential to minimize the likelihood of contamination of the control communities, while ensuring that the intervention is fully implemented in intervention communities. Key leaders of control communities will be aware of the project and its requirements,

Table 2: Baseline Comparisons Between Intervention (“I”) & Control (“C”) Communities

Measure	I	C
**2000 Total Population** Mean	15,814.25	13,927.50
Standard Deviation	12,446.06	9,824.09
**2000 Percent White** Mean Proportion	88.38%	88.74%
**1998 Percent Enrolled in Free/Reduced Lunch** Mean Proportion	26.71%	33.78%
**1998 Percent Unemployed** Mean Proportion	4%	3.09%
**Implementation Score (All Respondents)** Mean	1.08	1.25
Standard Deviation	0.79	0.97
**Implementation Score (Prevention Leaders)** Mean	1.58	2.08
Standard Deviation	0.10	0.10

Measure*	I	C
**Lifetime Alcohol Use** Mean Prevalence	0.51	0.52
**30 Day Alcohol Use** Mean Prevalence	0.27	0.28
**Lifetime Cigarette Use** Mean Prevalence	0.41	0.45
**30 Day Cigarette Use** Mean Prevalence	0.14	0.17
**Lifetime Marijuana Use** Mean Prevalence	0.18	0.22
**Past Year Delinquency** Mean Prevalence	0.21	0.23
**Total Risk** Mean	-0.03	0.01
Standard Deviation	0.57	0.58
**Total Protection** Mean	-0.03	-0.07
Standard Deviation	0.64	0.62

*Eighth Grade Students

but will not be oriented to, or provided training in, risk- and protective-focused prevention. They will not receive training or technical assistance in methods for forming a successful community board, conducting a community risk and resource assessment, using epidemiological data to prioritize specific risk and protective factors for preventive intervention, or selecting and implementing tested, effective interventions in mutually reinforcing ways throughout the community. Findings from the current Diffusion project indicate that coherent training and ongoing technical assistance are essential to the successful implementation and institutionalization of risk- and protective-focused prevention as a community-wide prevention strategy.

Risk of contamination will be reduced by the fact that all experimental and control communities are separated by at least 40 miles. In the intervention communities, newspapers, posters, flyers and radio will be the primary media used to educate, recruit and involve local community residents in prevention

activities. These channels are unlikely to contaminate control communities. While some control and intervention communities fall within the same television media markets, the distance between communities will prohibit exposure to the mobilization process and other intervention components used in intervention communities. Exposure to TV alone is unlikely to affect significantly the results of the project. Studies of the impact of mass media interventions to reduce substance use have generally concluded that media interventions alone, without associated community mobilization, policy or programmatic interventions, are not effective at reducing adolescent substance use and related problem behaviors (e.g., Holder & Treno, 1997; Korhonen et al., 1998). Finally, as described later, we will measure the active ingredients of the CTC intervention (community mobilization, implementation of risk- and protective-focused prevention planning, collaboration, and use of evidence-based prevention activities) in both experimental and control communities to identify possible contaminating influences. This will allow any observed contamination to be taken into account in analyses of intervention effects.

Figure 1. Study Design

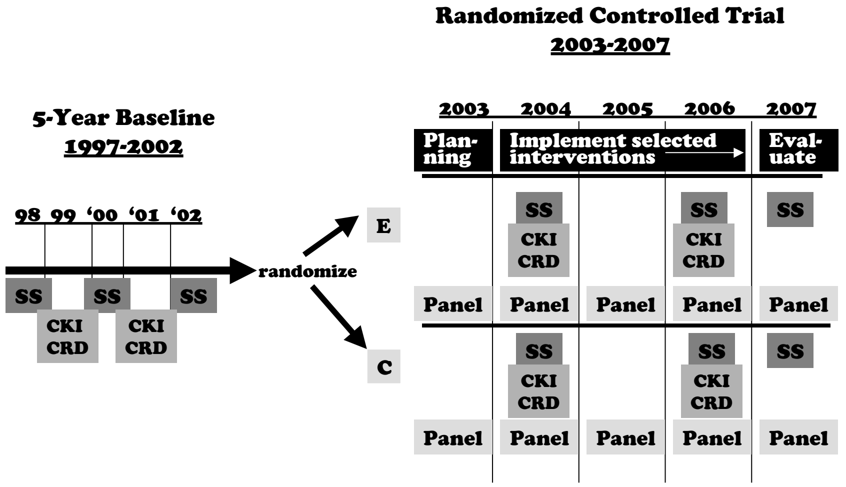

Study Design

The design of the proposed research project is presented in Figure 1, above.

Community youth samples. Evaluation of the impact of the CTC intervention on youth outcomes (Aim 1) will include comparisons between the intervention and control communities on trends in risk, protection and drug use and related problem behaviors assessed through repeated cross-sectional surveys of all consenting 6th, 8th, 10th, and 12th grade public school students in each community conducted in 2004, 2006, and 2007 (SS in Figure 1). Comparisons also will be made between the intervention and control communities on individual student-level changes in risk, protection, and initiation of substance use, delinquency, and violence though annual surveys of a longitudinal panel of 200 fourth grade students in each community recruited in the first year of the project and followed throughout the project (Panel in Figure 1).

Community key informant samples. Evaluation of CTC's impact on community prevention service systems (Aims 2-4) will include comparisons between the intervention and control communities on measures of collaboration, use of epidemiological data to guide planning and resource allocation decisions, and selection of tested prevention strategies. Data on these measures will be obtained through interviews with key informants in each community conducted in 2004 and 2006 (CKI in Figure 1).

Community prevention policy and program samples. Information on the types, quality, and scope of prevention activities consistent with effective interventions for elementary and middle school-aged youths and their families (Aims 3 and 4) will be obtained through structured interviews with prevention service providers and program directors, also conducted in 2004 and 2006 (CRD in Figure 1).

Strengths of the proposed study design. Conducting the proposed research with 12 pairs of communities participating in the Diffusion project provides several distinct advantages. These include: 1) three waves of baseline data measuring trends in risk, protection, and adolescent substance use, delinquency, violence, and related problems from student surveys conducted in each community prior to the start of the proposed study; 2) two waves of baseline data on each community's prevention planning approach including the degree of implementation of risk- and protective-focused prevention collected prior to the start of the proposed study; 3) two waves of baseline data on the number, types, and scope of science-based prevention strategies within each community collected prior to the start of the proposed study; and 4) a five-year history of collaboration between these communities and the researchers. Another strength of the proposed design is the combination of repeated cross-sectional surveys of students in four different grade levels with a longitudinal panel of students followed and surveyed annually for the five years of the project. Using the two survey methods, which provide complementary approaches to assessing intervention impact on risk, protection, and substance use and related problem behaviors, addresses different threats to the internal validity of study findings (Salonen et al., 1986; Wagner et al., 1991). Finally, the two assessment methods focus primarily on the age group

affected most directly by the prevention strategies implemented as a result of the CTC intervention, but also allow evaluation of the impact of broader community policy and norm changes on the prevalence of problem behaviors among older youths.

Measurement Methods, Schedules, and Instruments:

Since 1988, the Social Development Research Group has maintained a centralized data collection unit with extensive experience in gathering data from general population and clinical samples. The unit has interviewers and supervisors with extensive experience in various aspects of data collection, including recruitment, locating, and mail, telephone, individual in-person, and school-based, group-administered surveys. Our data collection unit has been successful in maintaining high completion rates, and in providing complete data with little missingness across items.

Cross-sectional surveys. In the spring of Project Years 2, 4, and 5, all 6th, 8th, 10th, and 12th grade public school classrooms within the intervention and comparison communities will complete the youth survey used in the Diffusion project. The cross-sectional surveys will be conducted in February of each academic year by the research subcontractor in each state using scanable forms developed and printed following specifications provided by the SDRG data collection unit. This process has been used successfully in the Diffusion project to conduct the cross-sectional surveys in that project's 41 communities.

Based on our experience in the Diffusion project, we anticipate obtaining usable data from a minimum of 70% of the public school students at each of the targeted grade levels in each community. The average single grade enrollment in 2000 across these 24 communities is 316 students, resulting in an average of approximately 221 useable surveys per grade per community. To ensure high participation rates, we have provided special monetary incentives for schools that achieve a 70% participation rate.

The effects of the elementary school interventions on risk and protective factors and early problem behavior initiation will be assessed by comparing cross-sectional sixth grade samples over time in experimental and control communities. The surveys at eighth grade (ages 13-14) provide measurement of the degree of spread of risk and problem behaviors by the end of middle school in intervention and control communities. The final eighth grade cohort (assessed in Project Year 5) will have received maximum exposure (four years) to the services implemented as a result of the CTC intervention, and the cumulative impact of the elementary and middle school interventions on the early initiation of drug use and related problem behaviors will be assessed at this grade level. The 10th grade surveys will allow detection of changes in risk, protection, and problem behaviors that result from the interventions provided during the middle school grades. Finally, the 12th grade surveys will provide a measure of community-level trends in adolescent outcomes measured at the end of high school and will allow assessment of the impact of the community-level interventions alone. This measurement design will allow for comparisons of trends across six cohorts for each age group over the five baseline years (collected in the Diffusion project) and the five years of the proposed study.

Longitudinal panel. Based on power analyses described later, a longitudinal panel of 200 students enrolled in the fourth grade in each community will be identified in the first year of the study and surveyed by SDRG data collection staff in each of the five years of the study. Representative samples of fourth grade students in each community will be recruited for participation in the longitudinal sample, and active parental consent will be obtained. Because we estimate that 280 students will be required to obtain 200 consenting panel members, the sample for the longitudinal panel will include the entire population of fourth grade public school students in the 16 communities of less than 20,000 total population in the study, and over 70% of the fourth grade students in six remaining communities. If children in this cohort in intervention communities are affected by the CTC intervention, the longitudinal panel sample should be sensitive to these effects.

Students participating in the panel who remain in the community through the second year of the project (when services resulting from the CTC intervention will be initiated in the intervention communities) will be identified and resurveyed in the spring of the second project year. Once students recruited into the panel sample have participated in the first two waves of data collection, we will track them and resurvey them annually for the remainder of the study regardless of whether they remain in the community or move elsewhere. Data obtained from the panel sample will provide a strong test of intervention effects (Salonen et al., 1986; Wagner et al., 1991). The students in the panel will receive the greatest exposure to the service and system-level changes produced by the CTC intervention. Moreover, the panel sample will allow analyses of individual characteristics, program exposure, and trajectories of risk, protection, and outcomes, and the relationship of these factors to intervention effects.

We expect to achieve an initial consent rate of 70% for the panel sample. We expect that 85% of these youth will remain in the community through the second year of the project and be retained in the panel. We expect that 90% of these youths will provide usable data in the fifth year of the study, yielding a final panel sample in each community of approximately 150 youths. Active youth and parental consent will be obtained from panel sample participants using consent-gathering techniques that have proven successful in our previous studies. The SDRG data collection staff has extensive experience obtaining high completion rates. The NIDA-funded Seattle Social Development Project (SSDP), which followed a panel of 808 students from age 10 to age 16 with extensive yearly interviews, achieved an interview completion rate of 95% (770) at age 16. Subsequent follow-up interviews of the SSDP panel have obtained completion rates of over 92% at ages 18, 21, and 24 (Battin et al., 1998; Hawkins et al., 1999).

Locating and retention. A variety of overlapping methods will be used to locate and track panel members over time, including a computer-based panel locator system, paid, on-line address database searches, telephone and reverse directory searches, and address correction mailings. Additionally, we will work with schools to track student transfers during and between school years. The panel locator database will contain information obtained during the initial consent and will include: (a) parent's Social Security Number; (b) names, addresses, and telephone numbers of multiple family members and friends of the parent and student who should know how to reach the subject; and (c) the names and addresses of the parent's employers and the schools the student has attended. If all these potential locator sources are exhausted, the parent's Social Security number will be submitted to a national on-line database service which can provide current address and telephone number information (as well and address and telephone histories), not only for the selected subjects, but also for neighboring households via reverse directories which provide telephone numbers based on address information alone. Finally, all correspondence will be sent "address correction requested" as a means of informing both telephone and on-line locating efforts.

Student survey administration. Both the cross-sectional and longitudinal surveys will be group administered during one classroom period and will take approximately 45 minutes to complete. The survey items and responses will be read to students in grades 4 and 5 so that reading ability will not be a source of bias. We will make every effort to ensure that subjects are comfortable and to assure them that their responses are confidential. We will further ensure that students do not have the opportunity to see each other's survey forms during administration. Where the students participating in the longitudinal panel constitute a sample rather than the entire grade population of students in study schools, they will be surveyed as a group in a school setting outside of their regular classrooms. As the panel moves into middle school, this procedure will be repeated. Students who move out of the study communities after the second year of the study will be tracked and surveyed individually using similar survey administration procedures and the same self-report, scannable survey instrument. These procedures will maintain parallel survey administration protocols across all participants.

We recognize that surveying students in public schools may underestimate prevalence of problem behavior by missing youths who do not attend school or may bias the estimate by missing students in private schools. However, this need not compromise the internal validity of the study if that bias is the same in both intervention and comparison communities. We will assess this threat in the cross-sectional surveys of 10th and 12th grade students by looking for differences in rates of school dropout in intervention versus control communities. Any differences observed can be addressed in analysis by adding dropout rate as a community-level covariate. Moreover, the longitudinal panel of fourth grade students will be followed regardless of their continued involvement in school.

Instrument. The Youth Survey will be used in the proposed study (See the Instrument and Item-Construct Dictionary in Appendix B, and Arthur et al., 2001; and Pollard et al., 1999 in Appendix A). This instrument measures risk and protective factor constructs in the community, school, family, and peer/individual domains as well as drug use, delinquency, violence, gang involvement, and depressive symptoms. This instrument has been used in the Diffusion project, providing three waves of cross-sectional baseline data for each community. For the proposed project, the Youth Survey will be modified for use in the fourth and fifth grade panel surveys to be developmentally appropriate. Pretesting and pilot testing of the Youth Survey for use with students in grade 5 has recently been completed for another study at SDRG and will inform modifications for the proposed study. A copy of that instrument (the International Youth Development Survey) is included in Appendix B.

Community Key Informant Interviews. To assess the level of community implementation of risk- and protective-focused prevention, telephone interviews will be conducted with key community leaders in both the intervention and comparison communities in projects years 2 and 4. The key informant

interviews will be administered by trained interviewers using computer-assisted telephone interviewing (CATI) technology. The use of CATI as a survey mode increases data quality by automating skip patterns, decreasing missingness, and eliminating out-of-range responses. A two-step reputational sampling method will be used to select approximately 15 key informants per community (Cohen et al., 1986; Wagner et al., 1991). The key informants will include experts in drug abuse and violence prevention, key community organizations and agencies involved in prevention programs, and local education, government, health, and business leaders. In the intervention communities, the Community Board will form part of the key informant sample but non-board members will also be surveyed. Special effort will be made to ensure representation of leaders of racial and ethnic minority groups and organizations. The key informant surveys will be conducted six months from the time of the Community Board surveys to reduce the burden of data collection activities.

Instrument. The community key informant (CKI) interview will use the protocol developed in the Diffusion project. It will include closed-ended questions to assess degree of implementation of risk- and protective-focused prevention and factors hypothesized to influence degree and rate of implementation. It will also include open-ended questions to allow respondents to describe their prevention approach and their experiences with implementation of risk- and protection-focused prevention. Specific constructs measured by the CKI interview include the respondents' ratings of their community's stage of implementation of the risk and protective prevention approach, their reports of interagency collaboration, and their reports regarding their organization's use of data to guide strategy selection, resource allocation, and program evaluation (see the Community Key Informant Interview and Item-Construct Dictionary in Appendix B).

Community Resource Documentation. The Community Resource Documentation (CRD) process will be used to measure the number and quality of promising prevention programs in the 24 study communities. The CRD data collection process developed for the Diffusion project is a combination of telephone interviews and mail surveys designed to document the prevention resources (policies, systems, coalitions, and laws and practices) that are in place within a community. All telephone surveys will be administered by trained interviewers using CATI technology. Mail surveys will be administered following Dillman's Total Design Method, which relies on personalization and multiple contacts to achieve high response rates (Dillman, 1991). The process begins by interviewing informants from social service agencies and prevention coalitions that have been previously identified in these communities through the Diffusion project. Mail surveys are also sent to the principal of each public elementary, middle, and high school in the community.

From the Agency, Coalition, and School Building surveys, a snowball sampling method is used to identify active prevention programs in each community. These community prevention programs are then contacted and interviewed. The focus of these interviews is to identify programs, policies, and practices that are consistent with those shown to be effective in controlled evaluation trials, and to assess the quality, scope, and target populations of these prevention activities.

The CRD data collection process will be conducted in intervention and control communities in Years 2 and 4. Respondent samples from the Diffusion project's CRD data collection efforts will be used to begin the CRD process in each community. New programs will be added as they are identified during the snowball sampling questions asked in each interview module. SDRG has developed an MS Access application that integrates the respondent tracking system and the computer-assisted telephone interviews to manage the survey sample and data during the data collection period. Respondents will be contacted first by mail and then by trained SDRG interviews by telephone to schedule and conduct the thirty-minute interviews.

Instruments. The Diffusion Consortium CRD survey instruments are included in Appendix B. The Agency interview collects information on the prevention staffing, funding, and the agency's collaborative prevention efforts, as well as a list of preventive programs sponsored by the agency. The Prevention Coalition interview measures any community mobilization strategies used by the community and the coalition's collaborative prevention efforts. School based prevention activities are assessed through the School Building survey administered to all public schools in the community serving K through 12th grade levels. School strategies measured in the school building survey include: (a) organizational change; (b) classroom organization, management and instructional strategies; (c) classroom curricula for social and emotional competence promotion; (d) school behavior management; and (e) school policies. The agency, coalition, and school building surveys also identify six specific types of prevention programs offered by the responding organization, and key informants for each program are identified. These include parent training, mentoring, tutoring, social competence

promotion, youth employment, and after school recreation programs. The identified program informants are then interviewed using a specific program interview module tailored to each program type. The screening criteria and quality measures for each program type used in the Diffusion project will be reviewed by the Scientific Advisory Board during the first six months of the Project to assure the validity of these criteria.

From the CRD interview process, we are able to calculate the numbers of programs of each type that are consistent with quality criteria in effective interventions, as well as measures of the scope (number and ages of youths served), dosage (total number of prevention hours offered), and overall exposure (scope x dosage) of each type of program in the community.

CTC Implementation Measures

Community board surveys. To monitor the community mobilization process, 20 CTC community board members in the intervention communities will be surveyed annually by phone using a revised Community Board Survey instrument initially developed by SDRG for the Oregon TOGETHER! project and the current CRD Coalition Questionnaire. The first survey will be conducted approximately 8 months after the CTC intervention is initiated. The CTC Board Survey will assess dimensions associated with developing and maintaining an effective community board (Gottlieb et al., 1993; Harachi Manger et al., 1992; Kumpfer et al., 1993; Moos, 1974; Prestby & Wandersman, 1985; Prestby et al., 1990): board membership, meeting schedule, meeting attendance, organizational structure, board cohesiveness, board leadership, task focus, planning products, use of data, perceived board cohesion, board efficacy, board member knowledge and skills, resources generated, leadership support, participation costs and benefits, member satisfaction, member recruitment and orientation, and continued involvement of key community leaders. The CTC Board Survey instrument has been tested with 35 community risk reduction planning boards, and scales have demonstrated good internal reliability (scale alpha coefficients ranging between .62 and .96) (Harachi Manger et al., 1992). (See Appendix B for Community Board survey instrument and CRD Coalition instruments.) This survey instrument will be expanded to incorporate constructs of community board functioning shown to predict higher implementation in recently completed studies evaluating the effectiveness of CTC boards (e.g., Greenberg et al., 1999) and other community prevention coalitions (e.g., Kegler et al., 1998).

Board activity reports and strategic plans. Monthly reports from each community coordinator to the project director will document the community board, task force, and staff activities and will include meeting minutes and attendance records. Copies of strategic plans will document community assessment, plans for implementing tested interventions, and evaluation plans.

Intervention implementation measures. In the intervention communities, monitoring of preventive intervention implementation will be an ongoing process to ensure that the interventions chosen by the community prevention boards achieve the integrity, scope, and intensity necessary to achieve the community's risk reduction and protective factor enhancement objectives. For each intervention, measures of implementation integrity and intensity will be obtained from the original testers of the intervention (see Appendix B for an example implementation checklist). Implementation standards will be specified for each intervention prior to implementation, though the specific implementation measures used in each community will vary depending on the interventions selected by the community board. These measures will be used to assist the intervention communities to monitor their progress in implementing the evidence-based interventions described in their community prevention plans. The feedback provided by these measures will be used to support the continuous quality improvement component of Phase V of the CTC intervention (Wandersman et al., 1998).

Community board training. Pre- and post-test questionnaires will be administered at each community board training session to assess knowledge gain, attitude change, and consumer satisfaction information. These questionnaires will be used to evaluate the quality of the CTC training programs provided by DRP.

Documentation of non-intervention community influences. Characteristics of each community in both the intervention and control conditions that might influence the community mobilization process or community prevalence of adolescent behavior problems will be obtained from the key informant survey described previously. Also, any major changes or events in the community that might affect program implementation (e.g., changes in community policies, layoffs by a major employer) or prevention efforts in the community will be documented using field staff logs and the key informant interviews.

Data Preparation and Management

Data management activities for all data will involve computerizing records, cleaning and insuring the quality and completeness of the data, providing basic output of frequencies and distributions of data elements, and providing documentation. SDRG's data managers have successfully completed this process for numerous data sets from multiple sites since 1994. Codebooks will include fully annotated questionnaires indicating variable names and values for response codes, an alphabetic variable dictionary that includes variable labels and response codes, program files for creating file architecture, and frequencies and relevant reliabilities for all variables and scales. Auxiliary manuals will include sources of data, cleaning protocols for out-of-range and logical inconsistencies, copies of command files that make permanent file modifications, and full records for created variables and scales with wording and coding of all items used, as well as compute statements for scale construction.

Analysis

We will apply our experience in creating and maintaining large data sets from multi-site studies to assure that the data obtained in the proposed study are of high quality. We begin our analyses by looking descriptively at the variables to be used and assessing the extent to which the assumptions of the proposed models are violated. Further, measurement models that have already been developed in prior studies will be evaluated in the data set using confirmatory techniques. For those constructs for which the measurement has not been previously validated, psychometric analyses will be conducted to provide strong grounds for conducting the proposed outcome analyses. We recognize the importance of addressing the nested data structure in the measurement as well as analysis of outcomes (James, 1982; Sirotnik, 1980). Psychometric analyses appropriate for the multilevel structure of the data will be used when evaluating the measurement of individual level variables (James, 1982; Kreft, 1993; Muthén, 1989, 1991, 1994; Raudenbush et al., 1991; Sirotnik, 1980).

The analysis of intervention effects on youth outcomes must incorporate the nesting of individuals within communities and the random assignment to conditions at the community level. Thus, the analyses need to incorporate multilevel models that take this nesting into account and provide unbiased estimates of standard errors (Bryk et al., 1996; Hox, 1998; Murray, 1998). The CTC intervention is expected to produce community-level changes in prevention service system characteristics, for example, increased collaboration among service providers and increased use of science-based prevention strategies. These changes in service systems characteristics are expected to produce student-level changes in risk and protective factors. These changes in risk and protective factors are expected, in turn, to affect changes in self reported adolescent drug use and related problem behaviors. The sections below describe both cross sectional and longitudinal comparisons of intervention and control communities.

Aim 1. To test the effectiveness of the CTC system in reducing risk, increasing protection and reducing problem behaviors.

<u>Cross-sectional cohort analyses</u>. Analyses for Aim 1 will begin by testing differences between the intervention and control communities for each grade each year on both specific and composite measures of risk and protective factors derived from the cross-sectional student surveys. These analyses will compare levels of specific risk and protective factors targeted by multiple intervention communities and composite measures of total risk and protection within each of four domains: community, school, family, and peer/individual. Multilevel General Linear Modeling techniques (Bryk & Raudenbush, 1992; Heck & Thomas, 2000; Murray, 1998), in which children are nested within communities, and communities nested within the matched pair, will be used. Analyses using the matched pairs design are proposed here because power analyses demonstrated that for most outcomes the matching, which assures that the intervention and control groups are similar on key variables at baseline, reduces the error variance in the dependent variables. Treatment will be modeled as a fixed effect predicting differences between communities within pairs. Alternately, school-level effects will be assessed through analyses nesting children within schools and schools within communities, using unmatched designs. Community-level covariates will be added to these models to reduce the community-level variance and, thus, increase the power of the analyses. For example, a community-level archival indicator of poverty such as those collected for the Diffusion project (e.g., unemployment, free and reduced lunch enrollments, etc.) will be entered as a time-varying covariate in some models to control for possible economic differences between communities. Community-level covariates will be limited to maintain the degrees of freedom available for testing the effects of intervention. Child-level covariates, such as gender, will be added to understand more about how the intervention affects different adolescents.

The analysis will then assess the effects of intervention on changes over time in communities' levels of risk and protective factors. These analyses will use growth curve models (Bryk & Raudenbush, 1987; Diggle et al., 1995) at the community level to examine the effect of intervention on slopes of risk and protection over time. These are flexible models that allow the use of individual covariates and community-level time-varying covariates. Standard multilevel models will be used, with four levels of nesting: children at level one nested within survey occasion at level two, nested within communities at level 3, and nested within pairs at level 4. In this model, growth curves exist at level 2, in which the community average scores at each time point are modeled as a function of an intercept and slope that represents change over time. Again, treatment is a fixed community-level effect that predicts the slope of the growth curve at level 3.

In the analyses above, intervention condition is used as a predictor of differences and changes in risk and protective factors. We will also assess the effect of the intervention by examining the relationship between community implementation of the CTC strategy and risk, protection, and adolescent behavior outcomes. As described earlier, the CKI interviews conducted in the second and fourth year of the study will provide data to create implementation scores for each community. These are ratings of the extent to which each community has advanced in implementing the CTC strategy on a scale of 1 to 5. Using implementation scores in analyses will allow assessment of whether variation in the degree to which the CTC intervention was implemented is related to differential outcomes. These analyses follow those above and will use the implementation score as an independent variable at the community level.

One of the major strengths of this study is that multiple years of baseline data are available for each of the communities. This allows us to posit growth models that go beyond describing differences in slopes by taking into account community-level slopes from before the intervention. Thus, the ability of the intervention to produce positive or negative trends within each community can be assessed. Community trends can be taken into account using Baysian estimates of the baseline slopes and intercepts within each community as covariates when predicting differences in the slope and intercept due to intervention (e.g., Bryk & Raudenbush, 1992). Use of the multiple baseline data points allows us to assess stability, or variance over time, of measures within each community.

These analyses will be extended to address Aim 4 to test the degree to which the use of tested programs in communities affects children's risk and protection exposure and behavior outcomes. The intervention is expected to increase collaboration and the use of effective interventions targeted at priority risk and protective factors. These variables can be used as community-level predictors to test whether they mediate the effect of the intervention on outcomes using the growth modeling approach.

Analyses of the effects of intervention on adolescent drug use and related behavior will be conducted using logit models that assess the effects of the intervention on the likelihood that children within each community engage in each of the targeted behaviors, initiation of alcohol, cigarette, and marijuana use, and incidence of delinquent and violent behavior. For example, we can begin analyses looking at the effects of intervention on reported alcohol use, a child level dependent variable at a single time point. A three level model in which children's reported alcohol use is treated as nested within community, and community is nested within the matched pair, will be used. The intervention will be considered a fixed effect at level 2. Again, in some models, school, instead of the matched pair, will be entered as a level. Analyses also will seek to assess intervention effects on growth in alcohol use across grades by combining grade level samples and entering grade as a covariate at the child level 1. This model can then be used for a longitudinal assessment of the effects of intervention on changes over time in the prevalence of alcohol use at the community level. Using HLM (Bryk & Raudenbush, 1992; Bryk et al., 1996) terminology and a logit link to model the binary outcome the model would be written:

Level-1, students	Probability of alcohol use $(Y = 1) = \varphi_{ijkp}$
	$\log(\varphi_{ijkp}/1-\varphi_{ijkp}) = \pi_{0jkp} + \pi_{1jkp}\text{Grade}$
Level-2, survey occasions	$\pi_{0jkp} = \beta_{00kp} + \beta_{01kp}\text{Year} + r_{0jkp}$
	$\pi_{1jkp} = \beta_{10kp}$
Level-3, communities	$\beta_{00kp} = \gamma_{000p} + u_{00kp}$
	$\beta_{01kp} = \gamma_{010p} + \gamma_{011p}\text{Treatment} + u_{01kp}$
	$\beta_{10kp} = \gamma_{100p} + \gamma_{101p}\text{Treatment}$
Level-4, pairs	$\gamma_{000p} = \zeta_{0000} + w_{000p}$
	$\gamma_{010p} = \zeta_{0100} + w_{010p}$
	$\gamma_{011p} = \zeta_{0110} + w_{011p}$

$$\gamma_{101p} = \zeta_{1010} + w_{101p}$$

This model postulates that the probability of an individual reporting alcohol use is a function of their grade and the average alcohol use at time j in community k within pair p. The growth portion of the model occurs at level 2, in which the proportion of youth within each community at a given time point using alcohol is modeled as a function of the initial alcohol use in that community and the slope related to the year of observation. At the community level, the effect of the intervention on change within communities and the relationship of grade to alcohol use is tested. Note that this is actually testing the interaction of the linear growth component with treatment and of the relationship of grade to outcomes with treatment. This is true because the model postulates that the slope of the growth curve varies as a function of treatment, the definition of an interaction. These four level models will be modeled in MLwiN (Rasbash et al., 2000), which allows for more than three levels of nesting.

Given that not all of the risk and protective factors are expected to be normally distributed, the assumption of normal and independently distributed errors within each level of nesting may not be appropriate (Feng et al., 2001). Permutation tests which do not make this assumption are available in group-randomized designs. The appropriateness of using these models will be examined. It is also possible to pose models other than growth models in testing the relationship of the intervention to change in outcomes. While we believe that growth models are reasonable, given that exposure will increase and services are expected to improve over the course of the study, thus improving outcomes, the fit of these models will be examined and other options considered if necessary.

Analyses of the longitudinal panel. Use of a longitudinal panel allows examination of how the intervention affects children as individuals rather than groups over time. We expect the potentially wide variation in individual-level growth curves will be hidden in group-level analyses. The individual-level longitudinal analyses will be powerful because they can account for differences between individuals in rates of change. These longitudinal analyses will involve the use of multilevel models and growth curve modeling as described above for risks and protection, but with the longitudinal sample the growth curves will be specified at the child level rather than the community level. The effects of treatment condition on average slopes within communities will be modeled at the community level. In order for these models to work, risk and protective factors must follow a growth pattern that can be identified and modeled. Previous work has shown that it is possible to specify individual level growth curves for risk and protective factors and that differences in their slopes can be predicted by involvement in intervention (Hawkins et al., in press). Effects of the intervention on substance use and problem behavior will be examined using three level logistic models in which engaging in substance use or problem behaviors by the eighth grade is modeled as a function of the intervention.

The longitudinal panel data also will allow examination of subgroups of children most affected by the intervention. For example, students experiencing moderate to high levels of risk exposure are more likely to be affected by intervention than students experiencing low levels of risk. We will assess whether initial levels of risk and protection are differentially related to changes in risk and protection in the intervention and control groups. This can be tested using a model in which baseline risk is a covariate predicting slopes of risk at the individual level, and then assessing the effect of the intervention on this relationship at the community level. Interactions between a variety of individual level effects and the intervention can be examined in this manner to better understand for whom the intervention has the largest effects. We have an ongoing consulting relationship with Dr. Steven Raudenbush who will serve as an advisory board member for the proposed project and will assist in the specification of multilevel analysis models.

Aim 2. To examine effects of CTC on levels of collaboration across agencies, groups and organizations in communities.

Aim 3. To examine effects of CTC on community use of epidemiologic data to guide prevention service system planning.

Aims two and three address the effects of the CTC intervention on community-level outcomes. Community outcome analyses will be conducted using single level models since the outcomes are viewed as characteristics of the community. Since the analyses for both aims are the same, they are described together. Both cross sectional and longitudinal analyses will be conducted to assess average differences and changes in collaboration or use of epidemiological data over time as they relate to the intervention.

Differences between treatment and control communities in community-level outcomes will be assessed using standard General Linear Models (GLMs). Community covariates will be used to reduce the error

variance of each model and help explain reasons for differences between communities. Further longitudinal analyses using growth curves will assess the effects of intervention on changes in collaboration and use of epidemiologic data for strategic prevention planning over time. These growth curves can be assessed using either multilevel GLM techniques or structural equation models. The advantage of using structural models is that they allow more efficient modeling of ordinal variables providing more accurate estimates of parameters and standard errors (Muthén et al., 1997). These analyses will also utilize the information available for these communities for the five years prior to intervention. One method, as described above, for looking at student outcomes, is to use the slopes and intercepts from the baseline period as predictors of the slopes during the intervention period. This allows for assessing whether the intervention is able to change trajectories across time.

As in the first aim, comparisons of treatment and control communities will be elaborated by separate analyses testing the extent to which CTC implementation relates to these community-level outcomes.

Aim 4. To assess the degree to which the use of tested effective programs in communities predicts changes in community-wide levels and trajectories of risk, protection, and drug use and related health and behavior outcomes.

Analyses for this aim are similar to those in Aim 1; the outcomes are measures of risk and protection and substance use and delinquent behavior at the student level and both cross-sectional models examining the relationships year by year and longitudinal models at the community level will be tested. Three- and four level HLM analyses will be used to assess these relationships as discussed above. We will begin by examining the overall relationship between total number, scope, dosage, and exposure of tested programs across all categories, as described above in the measures section, to composite measures of risk, protection, and outcomes. We will then elaborate these tests by examining the number, scope, dosage, and exposure of specific types of prevention programs (e.g., parenting skills training) and their relationship to changes in specific risk and protective factors (e.g., family management problems, parent-child bonding, etc.). We will also elaborate these tests by examining these relationships at the school building, rather than community level. The use of continuous and ordinal level predictors does not affect the specification of the multilevel models described above.

Given a relationship between these community-level outcomes and student outcomes and an effect of the intervention on student outcomes in Aim 1, these analyses will be further elaborated to examine the CTC framework. Specifically the framework posits that the intervention will positively affect youths through its impact on community prevention service system characteristics such as use of science-based strategies. We propose to examine this aspect of the framework by expanding on the results of the above analyses and testing a mediation model that examines the extent to which the community-level outcomes account for all or most of the effect of treatment on students. This can be examined by entering the community outcomes into the final models from Aim 1 as predictors at the community level. If the intervention is affecting students solely through programmatic effects at the community level, the treatment effect should become zero in this model. This mediation model can then be expanded with a longitudinal model in which community-level outcomes predict changes in student outcomes over the course of the study. These relationships will be examined in both the cross sectional data and in the longitudinal panel. Although theoretically more complex, these analyses are relatively simple expansions of the models used to assess Aim 1.

Power

The proposed study builds on a descriptive study that provides baseline data for all proposed sites. This allows us to use parameter estimates from the sample under study to estimate the power of the proposed study to detect effects. Analyses have been conducted using data from each study community collected in 2000. Thus, our power analyses provide very accurate assessments of the differences between intervention and control groups that can be observed with adequate power.

The power analyses for adolescent outcomes assume that children are nested within communities. A three level structure in which students are nested within schools nested within communities is also possible. Separate three level power analyses were conducted and only small differences were found for the more complex design. Further, all analyses assumed continuously varying, normally distributed outcomes. While this assumption is violated for binary outcomes, simulations have found that, given at least 30 members per group, the Central Limit Theorem still holds and the analyses will still provide adequate parameter estimates (Hannan & Murray, 1996).

Power was estimated for analyses using a simple comparison of the experimental and control groups, then for analyses using a matched pair design, and finally for analyses using matched pairs in which

1998 average community levels of each outcome is included as a community-level baseline covariate. While the longitudinal data we have for the communities does not yet extend for more than two years, including the 1998 scores allows an assessment of the ability to detect how the intervention is related to change in communities rather than just differences in community means. The principal task in power analyses is to find an estimate of the variance of the treatment effect, $\sigma\Delta$. Then t-statistics can be calculated and formulas can be applied to find the power for any given treatment effect. As described in Appendix G, the power of the proposed study was calculated by first finding the standard error of the treatment effects (Bryk & Raudenbush, 1992; Murray, 1998; Raudenbush, 1997; Raudenbush et al., 2000) and then using these standard errors to find the detectable treatment effect with a power of .80.

Estimates of $\sigma\Delta$ were found by using data from the study communities. Since the community is the level of random assignment, the sample size is 24 (Murray, 1998). Analyses were done using only eighth grade students since that is the grade expected to be most directly affected by the intervention. For the sake of brevity, two representative risk factors and the most relevant behavioral outcomes for the targeted age group were used in the power analyses.

We found that in most cases use of the matched pairs design reduced the community-level variance enough to result in an increase in power even when taking into account the loss of degrees of freedom. Further, the addition of 1998 baseline outcome measures as community-level covariates generally resulted in large decreases in community-level variance resulting in further increases in power. The power of the matched pairs analyses with community-level covariates is reported in Table 3. For a full description of these analyses, see Appendix G.

Results of these analyses indicate that this study has adequate power to detect differences in student's risk and protective factors equivalent to a small effect size of .25. Effect sizes of .2 or larger are generally considered to be small but of practical significance, while effect sizes of .50 and above are generally considered large in social science research (Cohen, 1977). The study has power to detect differences of 12% in rates of lifetime use of alcohol, 7% in rates of lifetime use of cigarettes, and 7% in rates of alcohol use during the past month (a 22, 16, and 25% reduction, respectively). Average prevalence rates for these variables are reported in Table 3 in the means column. These effects are at the low end of the range of what has been demonstrated by other successful community-level interventions which have found reductions in initiation of alcohol and cigarette use in the eighth grade of between 20 and 40% (Pentz et al., 1997; Perry et al., 2000; Perry et al., 1996). The detectable difference for risk factors is based on the 4-point scale measuring these constructs.

The power reported here for the child level outcomes is expected to be the minimum that is observed in the longitudinal analyses both at the community level and at the child level in the longitudinal panel.

Secondly, an examination of the power of the study to detect changes in implementation scores from three or less to above three was conducted using non-parametric techniques. These analyses found the study will have adequate power to detect a significant difference if six more experimental communities than controls achieve the desired implementation result.

Table 3: Detectable Differences in Eighth Grade Student Outcomes Between Intervention and Control Groups

Outcome	M (SD)	Power 0.80	Effect Size/ % Reduction*
Total Risk Score	-0.02 (.58)	0.120	.21
Total Protection Score	-0.05 (.63)	0.184	.28
Favorable Attitudes Towards Drug Use	1.60 (.75)	0.184	.25
Perceived Risk of Drug Use	1.85 (.76)	0.131	.18
Friends' Use of Drugs	0.90 (1.06)	0.262	.25
Lifetime Cigarette Use	0.43	0.078	18%
Lifetime Alcohol Use	0.52	0.130	25%
30 Day Alcohol Use	0.27	0.078	29%
Violence	0.15	0.058	37%
Delinquency	0.22	0.075	35%

* $\beta = 0.80$

e. Human Subjects

Subject Characteristics

Survey data on risk and protective factors and drug use and related problem behaviors will be collected in Project Years 2, 4, and 5 from all consenting 6th, 8th, 10th, and 12th grade public school students, and from annual surveys of a representative sample of 200 students in the 4th grade panel recruited in Project Year 01, in each participating community. These samples have been selected to assess the impact of the intervention on children in the age range targeted by the intervention. Data on characteristics of the community and community prevention services will also be collected in Project Years 2 and 4 from a sample of 15 community leaders and from approximately 92 prevention service providers in each community, and in all years from approximately 20 adult members of the Community Prevention Board created in each of the 12 intervention communities.

Racial and Ethnic Composition of the Sample

Table 4 is based on numbers of students of different races and ethnic backgrounds who currently reside in the project communities. Although the exact number and characteristics of the students who will be exposed to the intervention and who will participate in the research cannot be provided at this time, the intervention design sets clear expectations regarding participation of community members from diverse backgrounds. The following assumptions were applied to 2000 Census estimates of the racial and ethnic composition of each collaborating community to derive estimates of the numbers of individuals of each major ethnic and racial group expected to participate in this study: (a) the minority composition of intervention participants will equal the minority composition of the communities sampled; (b) two-thirds of the 5th-8th grade students and families in each intervention community with enrollment of over 200 per grade, and all of the 5th-8th grade students and families in each intervention community with enrollment of 200 or less per grade, will be exposed to elements of the intervention; and (c) in each community, 200 panel members plus 225 6th, 8th, 10th, and 12th graders will be surveyed, with a new 6th grade sample and an accretion sample of 20% of 8th, 10th and 12th graders added in Year 4, and a new sample of 225 students in each grade surveyed added in Year 5. The estimates are presented in Table 4.

TABLE 4: Expected Ethnic Composition of Study Participants

	Total expected for intervention	Total youths surveyed (Grades 4-12)
White	20,266	39,181
African American	427	916
Hispanic	2087	4379
Asian/Pacific Islander	255	515
American Indian/Native Alaskan	176	344

Recruitment and Consent Procedures

For students participating only in anonymous cross-sectional surveys from whom no identifying information will be collected, passive parental consent procedures will be used unless active consent is required by state law. Because these student survey data are used by school districts to evaluate their prevention programs as school educational innovations, passive consent procedures are appropriate. Parents will be sent a letter describing the purpose and benefits of the study, what will be asked of their child (completion of an anonymous, confidential questionnaire administered during class), and the content of the questionnaire, and stating that their child's participation is voluntary. If parents do not want their child to participate, they will be asked to contact their child's school or send back a form included with the letter. Letters will be sent within a time frame that will allow adequate time for parents to respond. Parents will also be provided with an SDRG data collection telephone number to call toll free to request additional information about the study.

Active parental consent will be obtained from the 200 students randomly selected and recruited in each community in Year 1 to comprise the longitudinal panels. After receiving a mailing describing the study, the parents of the selected students will be contacted by the data collection field supervisor in each community. The field supervisors will receive training in obtaining consent without pressuring the respondents. The purpose and benefits of the study, how children were selected (as randomly selected members of their grade cohort), what will be asked of their child (completion of a series of five

confidential questionnaires, one each year for five years administered during one class period in school), the content of the questionnaire, and the procedures for maintaining confidentiality will be explained. As in the cross-sectional sample, parents of children selected for the panel sample will be informed that their child's participation is voluntary. Parents who agree to their child's participation will be asked to sign an informed consent form. Once consent has been received, the child's name and a confidential identifier will be entered into a secure database. Surveys with the identifiers on them and a tear-off cover sheet with the child's name will be prepared and distributed to students in the panel sample during the survey administration. To protect further the confidentiality of survey responses, the investigators will obtain a federal Certificate of Confidentiality for this project.

Community leader, prevention service provider, and community prevention board member interviews will be confidential, but cannot be anonymous. Potential adult participants will initially be contacted by letter and informed of the purpose and benefits of the study and the procedures involved, and informed that the surveys are confidential and that participation is voluntary. At the time of the interview, consent will be obtained over the phone and respondents will be reminded that the survey is confidential, their names will not be linked to their survey, and that their participation is voluntary. Archival records (e.g., police records, emergency room records, health department records, and school records) will be obtained on an aggregate (i.e., community) level with no information that identifies individuals.

Potential Risks

Students could potentially experience some stress or discomfort from filling out the CTC Youth Survey questionnaire due to disclosure of drug use, aggressive behavior, or other personal information pertaining to risk factors. Community leaders, prevention service providers, and Community Prevention Board members could potentially experience some distress if their confidentiality is violated and their views of their community are made public. While no questions will be asked on the latter three surveys about individual behaviors that might be sensitive, opinions and attitudes toward the community, the community prevention board, and community prevention providers could potentially have political or interpersonal ramifications.

Minimization of Risks

The potential for discomfort caused by the student surveys is reduced considerably because most of the surveys are anonymous. Most surveys will be administered to entire classrooms, and no identifying information will be obtained from the students. However, the surveys of the longitudinal panel of students are not anonymous. The potential for discomfort among students in the panel sample will be reduced by strict measures to ensure confidentiality. The students' names will never be placed on the survey questionnaires themselves. Instead, their names will appear only on a cover sheet that will be torn off by research staff when the surveys are passed out during administration, and a coded identifier will be printed on the survey pages. The investigators will obtain a federal Certificate of Confidentiality to ensure further protection of all respondents' confidentiality.

All surveys will be administered following standard survey administration procedures developed by SDRG project staff. At the time questionnaires are administered, measures to ensure confidentiality will be explained and students will be informed that their participation is voluntary. Students will also be given the opportunity to ask questions before and after the survey is administered. Completed survey forms will be placed in large envelopes in each classroom for transport to organizations that will scan the survey forms into electronic data files. The competed cross sectional surveys will be kept in locked cabinets at each collaborating state research contractor until they are destroyed. Completed surveys from the longitudinal panel will be kept in locked cabinets at SDRG until destroyed. To ensure confidentiality for the community leader, prevention provider, and Community Prevention Board interviews, identification codes will be used rather than names. Most of these interviews will be conducted over the phone by research staff at the central site (SDRG, Seattle) using computer-assisted telephone interviewing stations, and the resulting electronic data files will be maintained in password protected directories within SDRG’s internal computer network. Mail surveys of school prevention programs and city policies will be kept in locked cabinets at SDRG until they are destroyed. In each case, lists of participants’ names will be kept in separate locked files apart from the survey data files.

Benefits

If CTC is found to be effective, the benefits from this study will outweigh the minimal potential for risks. Participating communities assigned to the intervention condition will be empowered to plan and

implement programs that will have an ongoing positive impact on the health and development of community residents. Risks for adolescent health and behavior problems will be reduced, protective factors promoting the healthy development of community members will be enhanced, and rates of alcohol, tobacco, and other drug use, violence, and crime will be reduced in the intervention communities. Participating states, communities assigned to the control condition, and society in general, will benefit from learning the effects of the CTC strategy. It will also be beneficial and important to learn that the CTC system does not produce the desired outcomes in communities, if it is not found to be effective.
