# Supplementary material for: Effect of the Communities That Care Prevention System on Adolescent Handgun Carrying: A Cluster-Randomized Clinical Trial
Source: JAMA Netw Open. 2023 Apr 6;6(4):e236699. doi: 10.1001/jamanetworkopen.2023.6699 (PMC10080373; doi:10.1001/jamanetworkopen.2023.6699)
Supplement: Supplement 2. — eTable 1. Findings of Primary Analysis 1 for Past-Year Handgun Carrying eTable 2. Findings of Primary Analysis 1 for Grade-Specific Past-Year Handgun Carrying eTable 3. Findings of Primary Analysis 2 for Cumulative Handgun Carrying eTable 4. Findings of Sensitivity Analysis 1 for Past-Year Handgun Carrying eTable 5. Findings of Sensitivity Analysis 2 for Past-Year Handgun Carrying eTable 6. Findings of Sensitivity Analysis 3 for Past-Year Handgun Carrying eTable 7. Findings of Sensitivity Analysis 4 for Past-Year Handgun Carrying eTable 8. Findings of Sensitivity Analysis 5 for Cumulative Handgun Carrying [file jamanetwopen-e236699-s002.pdf]

Supplemental Online Content

Rowhani-Rahbar A, Oesterle S, Gause EL, et al. Effect of the Communities That Care Prevention System on adolescent handgun carrying: a cluster-randomized clinical trial. *JAMA Netw Open*. 2023;6(4):e236699. doi:10.1001/jamanetworkopen.2023.6699

eTable 1. Findings of Primary Analysis 1 for Past-Year Handgun Carrying

eTable 2. Findings of Primary Analysis 1 for Grade-Specific Past-Year Handgun Carrying

eTable 3. Findings of Primary Analysis 2 for Cumulative Handgun Carrying

eTable 4. Findings of Sensitivity Analysis 1 for Past-Year Handgun Carrying

eTable 5. Findings of Sensitivity Analysis 2 for Past-Year Handgun Carrying

eTable 6. Findings of Sensitivity Analysis 3 for Past-Year Handgun Carrying

eTable 7. Findings of Sensitivity Analysis 4 for Past-Year Handgun Carrying

eTable 8. Findings of Sensitivity Analysis 5 for Cumulative Handgun Carrying

This supplemental material has been provided by the authors to give readers additional information about their work.

© 2023 Rowhani-Rahbar A et al. *JAMA Network Open.*

eTable 1. Findings of Primary Analysis 1 for Past-Year Handgun Carrying

Variable	Adjusted OR (95% CI)
CTC intervention	0.73 (0.65, 0.82)
Wave	
Grade 7 (vs. Grade 6)	0.64 (0.53, 0.79)
Grade 8 (vs. Grade 6)	0.86 (0.70, 1.06)
Grade 9 (vs. Grade 6)	0.76 (0.57, 1.02)
Grade 10 (vs. Grade 6)	0.80 (0.62, 1.03)
Grade 12 (vs. Grade 6)	0.80 (0.63, 1.03)
Female	0.13 (0.10, 0.17)
White	0.82 (0.58, 1.17)
Hispanic	1.00 (0.71, 1.40)
Parents college educated	0.95 (0.76, 1.18)
Percent on free and reduced lunch	1.01 (0.99, 1.02)
School enrollment	1.00 (1.00, 1.00)
Age in Grade 5	1.48 (1.08, 2.04)
Religious service attendance in Grade 5	0.96 (0.77, 1.20)
Rebellious scale score in Grade 5	1.83 (1.51, 2.21)
Community pair fixed effects	Included

OR: Odds Ratio; CI: Confidence Interval; CTC: Communities That Care

© 2023 Rowhani-Rahbar A et al. *JAMA Network Open.*

eTable 2. Findings of Primary Analysis 1 for Grade-Specific Past-Year Handgun Carrying

**Variable**	**Adjusted OR (95% CI)**
CTC intervention	0.79 (0.59, 1.06)
Wave	
Grade 7 (vs. Grade 6)	0.68 (0.50, 0.93)
Grade 8 (vs. Grade 6)	1.00 (0.77, 1.30)
Grade 9 (vs. Grade 6)	0.83 (0.55, 1.27)
Grade 10 (vs. Grade 6)	0.77 (0.54, 1.11)
Grade 12 (vs. Grade 6)	0.77 (0.56, 1.04)
CTC*Wave	
Grade 7*CTC	0.89 (0.59, 1.34)
Grade 8*CTC	0.73 (0.49, 1.09)
Grade 9*CTC	0.82 (0.47, 1.43)
Grade 10*CTC	1.06 (0.62, 1.80)
Grade 12*CTC	1.11 (0.67, 1.76)
Female	0.13 (0.10, 0.17)
White	0.82 (0.58, 1.17)
Hispanic	1.00 (0.70, 1.40)
Parents college educated	0.95 (0.76, 1.18)
Percent on free and reduced lunch	1.01 (0.99, 1.02)
School enrollment	1.00 (1.00, 1.00)
Age in Grade 5	1.48 (1.08, 2.41)
Religious service attendance in Grade 5	0.96 (0.77, 1.20)
Rebellious scale score in Grade 5	1.83 (1.51, 2.21)
Community pair fixed effects	Included

OR: Odds Ratio; CI: Confidence Interval; CTC: Communities That Care

© 2023 Rowhani-Rahbar A et al. *JAMA Network Open.*

eTable 3. Findings of Primary Analysis 2 for Cumulative Handgun Carrying

Variable	Adjusted OR (95% CI)
CTC intervention	0.76 (0.70, 0.84)
Female	0.23 (0.19, 0.28)
White	0.85 (0.70, 1.08)
Hispanic	0.96 (0.74, 1.26)
Parents college educated	0.92 (0.76, 1.13)
Percent on free and reduced lunch	1.01 (0.99, 1.01)
School enrollment	1.00 (1.00, 1.00)
Age in Grade 5	1.34 (1.07, 1.68)
Religious service attendance in Grade 5	0.99 (0.83, 1.18)
Rebellious scale score in Grade 5	1.56 (1.32, 1.84)
Community pair fixed effects	Included

OR: Odds Ratio; CI: Confidence Interval; CTC: Communities That Care

© 2023 Rowhani-Rahbar A et al. *JAMA Network Open.*

eTable 4. Findings of Sensitivity Analysis 1 for Past-Year Handgun Carrying

**Variable**	**Adjusted OR (95% CI)**
CTC intervention	0.76 (0.66, 0.86)
Female	0.18 (0.14, 0.25)
White	0.93 (0.64, 1.33)
Hispanic	1.08 (0.73, 1.61)
Parents college educated	0.97 (0.78, 1.21)
Percent on free and reduced lunch	1.00 (0.99, 1.02)
School enrollment	1.00 (1.00, 1.00)
Handgun carrying in Grade 6	9.53 (6.68, 13.60)
Age in Grade 5	1.27 (0.89, 1.80)
Religious in Grade 5	0.96 (0.78, 1.17)
Rebellious scale score in Grade 5	1.60 (1.33, 1.93)
Wave fixed effects	Included
Community pair fixed effects	Included

OR: Odds Ratio; CI: Confidence Interval; CTC: Communities That Care
Note. In this analysis, we shifted the analysis forward by one year so Grade 6 could be used as the baseline.

© 2023 Rowhani-Rahbar A et al. *JAMA Network Open.*

eTable 5. Findings of Sensitivity Analysis 2 for Past-Year Handgun Carrying

Variable	Adjusted OR (95% CI)
CTC intervention	0.77 (0.66, 0.89)
Female	0.18 (0.13, 0.25)
White	0.83 (0.58, 1.19)
Hispanic	0.89 (0.57, 1.40)
Parents college educated	0.93 (0.73, 1.19)
Percent on free and reduced lunch	1.00 (0.99, 1.02)
School enrollment	1.00 (1.00, 1.00)
Age in Grade 5	1.31 (0.90, 1.91)
Religious service attendance in Grade 5	0.92 (0.76, 1.13)
Rebellious scale score in Grade 5	1.62 (1.31, 2.02)
Wave fixed effects	Included
Community pair fixed effects	Included

OR: Odds Ratio; CI: Confidence Interval; CTC: Communities That Care
Note. In this analysis, we repeated Sensitivity Analyses 1 only among youth who did not report handgun carrying in Grade 6.

© 2023 Rowhani-Rahbar A et al. *JAMA Network Open.*

eTable 6. Findings of Sensitivity Analysis 3 for Past-Year Handgun Carrying

Variable	Adjusted OR (95% CI)
CTC intervention	0.79 (0.65, 0.91)
Female	0.15 (0.11, 0.19)
White	0.83 (0.58, 1.19)
Hispanic	0.96 (0.69, 1.35)
Parents college educated	0.98 (0.77, 1.26)
Percent on free and reduced lunch	1.01 (0.99, 1.03)
School enrollment	1.00 (1.00, 1.00)
Age in Grade 5	1.52 (1.09, 2.10)
Religious service attendance in Grade 5	0.99 (0.78, 1.26)
Rebellious scale score in Grade 5	1.45 (1.25, 1.79)
Delinquency scale score in Grade 5	1.36 (1.14, 1.63)
Wave fixed effects	Included
Community pair fixed effects	Included

OR: Odds Ratio; CI: Confidence Interval; CTC: Communities That Care
Note. In this analysis, we adjusted for delinquency in Grade 5 as a proxy measure for handgun carrying.

© 2023 Rowhani-Rahbar A et al. *JAMA Network Open.*

eTable 7. Findings of Sensitivity Analysis 4 for Past-Year Handgun Carrying

Variable	Adjusted OR (95% CI)
CTC intervention	0.80 (0.67, 0.96)
Female	0.15 (0.11, 0.20)
White	0.81 (0.51, 1.30)
Hispanic	1.04 (0.57, 1.89)
Parents college educated	0.97 (0.68, 1.39)
Percent on free and reduced lunch	1.00 (0.98, 1.02)
School enrollment	1.00 (1.00, 1.00)
Age in Grade 5	1.35 (0.94, 1.94)
Religious service attendance in Grade 5	0.86 (0.64, 1.10)
Rebellious scale score in Grade 5	1.60 (1.20, 2.29)
Wave fixed effects	Included
Community pair fixed effects	Included

OR: Odds Ratio; CI: Confidence Interval; CTC: Communities That Care
Note. In this analysis, we repeated Sensitivity Analysis 3 only among youth who did not report delinquency in Grade 5.

© 2023 Rowhani-Rahbar A et al. *JAMA Network Open.*

eTable 8. Findings of Sensitivity Analysis 5 for Cumulative Handgun Carrying

Variable	Adjusted OR (95% CI)
CTC intervention	0.76 (0.70, 0.84)
Female	0.23 (1.89, 0.28)
White	0.85 (0.67, 1.08)
Hispanic	0.96 (0.74, 1.26)
Parents college educated	0.92 (0.76, 1.13)
Percent on free and reduced lunch	1.00 (0.99, 1.01)
School enrollment	1.00 (1.00, 1.00)
Age in Grade 5	1.34 (1.07, 1.68)
Religious service attendance in Grade 5	0.99 (0.83, 1.18)
Rebellious scale score in Grade 5	1.56 (1.32, 1.84)
Community pair fixed effects	Included

OR: Odds Ratio; CI: Confidence Interval; CTC: Communities That Care
Note. In this analysis, we repeated Primary Analysis 2 only among youth who did not report handgun carrying in Grade 6.

© 2023 Rowhani-Rahbar A et al. *JAMA Network Open.*
