# Supplementary material for: Effect of the Communities That Care Prevention System on Adolescent Handgun Carrying: A Cluster-Randomized Clinical Trial
Source: JAMA Netw Open. 2023 Apr 6;6(4):e236699. doi: 10.1001/jamanetworkopen.2023.6699 (PMC10080373; doi:10.1001/jamanetworkopen.2023.6699)
Supplement: Supplement 3. — Data Sharing Statement [file jamanetwopen-e236699-s003.pdf]

Data Sharing Statement

Rowhani-Rahbar. Effect of the Communities That Care Prevention System on Adolescent Handgun Carrying. *JAMA Netw Open*. Published April 06, 2023.
doi:10.1001/jamanetworkopen.2023.6699

Data
Data available: Yes
Data types: Deidentified participant data, Data dictionary
How to access data: Please see the University of Washington's Social Development Research Group Fair Data Use Agreement form. Please contact the corresponding author to access this form.
When available: With publication
